# Thymosin beta-4 regulates activation of hepatic stellate cells via hedgehog signaling

**DOI:** 10.1038/s41598-017-03782-x

**Published:** 2017-06-19

**Authors:** Jieun Kim, Jeongeun Hyun, Sihyung Wang, Chanbin Lee, Jae-Wook Lee, Eun-Yi Moon, Heejae Cha, Anna Mae Diehl, Youngmi Jung

**Affiliations:** 1 Department of Integrated Biological Science, College of Natural Science, Pusan National University, 63-2 Pusandaehak-ro, Geumjeong-gu, Pusan, 46241 Republic of Korea; 2 Department of Biological Sciences, College of Natural Science, Pusan National University, 63-2 Pusandaehak-ro, Geumjeong-gu, Pusan, 46241 Republic of Korea; 3 Department of Bioscience and Biotechnology, Sejong University, 209 Neungdong-ro, Gwangjin-gu, Seoul, 05006 Republic of Korea; 4 Department of Parasitology and Genetics, Kosin University College of Medicine, 262 Gamcheon-ro, Seogu, Pusan, 49267 Republic of Korea; 5 Division of Gastroenterology, Department of Medicine, Duke University, 595 LaSalle Street, Durham, NC, 27710 USA

## Abstract

The molecular mechanisms of thymosin beta-4 (TB4) involved in regulating hepatic stellate cell (HSC) functions remain unclear. Therefore, we hypothesize that TB4 influences HSC activation through hedgehog (Hh) pathway. HSC functions declined in a TB4 siRNA-treated LX-2. *TB4* suppression down-regulated both integrin linked kinase (*ILK*), an activator of smoothened, and phosphorylated glycogen synthase kinase 3 beta (*pGSK-3B*), an inactive form of GSK-3B degrading glioblastoma 2 (GLI2), followed by the decreased expression of both smoothened and *GLI2*. A TB4 CRISPR also blocked the activation of primary HSCs, with decreased expression of smoothened, *GLI2* and *ILK* compared with cells transfected with nontargeting control CRISPR. Double immunostaining and an immunoprecipitation assay revealed that TB4 interacted with either smoothened at the cytoplasm or GLI2 at the nucleus in LX-2. Smoothened suppression in primary HSCs using a Hh antagonist or adenovirus transduction decreased *TB4* expression with the reduced activation of HSCs. Tb4-overexpressing transgenic mice treated with CCl_4_ were susceptible to the development hepatic fibrosis with higher levels of ILK, pGSK3b, and Hh activity, as compared with wild-type mice. These findings demonstrate that TB4 regulates HSC activation by influencing the activity of Smoothened and GLI2, suggesting TB4 as a novel therapeutic target in liver disease.

## Introduction

Chronic liver disease is associated with substantial mortality and morbidity worldwide^1^. Liver fibrosis, a common pathological feature of most chronic liver diseases, occurs in response to liver damage^2^. Severe and/or chronic damage accelerates liver fibrosis, which distorts the normal functions and structure of the liver by occupying parenchymal areas and replacing hepatocytes, culminating in cirrhosis. Liver cirrhosis results from dysregulation of the repair response to liver injury^3^. Hepatic stellate cells (HSCs) are key cells involved in the pathogenesis of liver fibrosis^4–6^. In healthy liver, HSCs are nonproliferative and exhibit a quiescent phenotype. Liver injury triggers transdifferentiation of quiescent HSCs, resulting in proliferative activated or myofibroblastic (MF) HSCs, and the activation of profibrogenic signaling factors, such as transforming growth factor beta (TGFB) and phosphatidylinositol-3-kinase^7, 8^. Excessive accumulation of MF-HSCs promotes liver fibrosis with increased inflammation, eventually leading to liver cirrhosis. Increased understanding of the molecular mechanisms regulating HSC activation is needed to develop therapeutic strategies to combat liver fibrogenesis.

Hedgehog (Hh) signaling plays important roles in liver development and pathogenesis, even cancer^9^. Hh signaling is initiated by the binding of Hh ligands, such as sonic Hh (SHH), indian Hh, and desert Hh, to the Hh receptor patched, activating smoothened (SMO), an effector of Hh signaling. This activated SMO in turn promotes the production of the transcriptionally active forms of the glioblastoma (GLI) family (GLI1, GLI2, and GLI3)^9, 10^. These activated GLIs accumulate in the nucleus and regulate the expression of Hh-target genes^9, 11^. Hh signaling is an essential pathway in HSC activation^12–15^. Growing evidence suggests that activation of the SMO-linked GLI2 pathway is a key event in liver fibrosis^16–19^. Treatment with a SMO antagonist (e.g., GDC-0449) reduced Hh expression by blocking the activation of GLI2 in activated HSCs and resulted in inactivation of HSCs, alleviating liver fibrosis^17, 18^. Therefore, interrupting SMO associated with GLI2 activation may be a valid strategy for controlling the activation of HSCs during liver fibrogenesis.

Thymosin beta-4 (TB4), an important G-actin sequestering protein, is the most abundant peptide of the beta-thymosin family^20^. Recent studies have described multiple biological functions of TB4, such as promoting wound healing, angiogenesis, and tissue regeneration^21–23^. However, the biological effects of TB4 in the liver are poorly understood. Furthermore, its potential role in liver fibrosis remains controversial^24^. In previous research, we reported that TB4 was a novel regulator of HSC activation^25^. We showed that endogenous levels of *TB4* were upregulated in fibrotic liver tissues of both patients with chronic liver disease and mice treated with carbon tetrachloride (CCl_4_), as compared to healthy livers. Moreover, TB4 was mainly expressed by activated HSCs, and knockdown of *TB4* in HSCs suppressed the activation of HSCs. However, the detailed mechanism underlying how TB4 regulates the activation of HSCs remains unknown.

In the absence of Hh signaling, GLI2-full length (FL) was shown to be phosphorylated by glycogen synthase kinase 3 beta (GSK3B), and phosphorylated GLI2 was subsequently ubiquitinated and degraded^9, 26–28^. In the presence of Hh, integrin linked kinase (ILK) phosphorylates GSK3B and forms phosphorylated GSK3B (pGSK3B), the inactive form of GSK3B. As a result, GLI2 phosphorylation by GSK3B is inhibited, proteolysis is blocked, and GLI2-FL is converted to transcriptionally the active form, GLI2-activator (A)^26–29^. Although SMO is also involved in production of GLIs-A, but the association of ILK-GSK3B with SMO in activating Glis is poorly understood. Previous research reported that ILK mediated Hh signaling by regulating the localization of SMO in the cilium^30^. It also demonstrated that TB4 stabilized ILK in cardiomyocyte and colon cancer cells and that overexpression of TB4 inactivated GSK3B by hyper-phosphorylating GSK3B^31–33^.

Hh signaling is a well-known regulator of HSC activation^12–15^. Thus, the present study investigated whether and how TB4 interacted with the Hh signaling pathway during HSC transdifferentiation using *in vitro* and *in vivo* systems. The results showed that TB4 was involved in activation of SMO by regulating ILK and GSK3B, contributing to the stabilization of GLI2. In addition, TB4 promoted the nuclear translocation of GLI2. Specific knockdown of *TB4* expression by siRNA or the clustered regularly interspaced short palindrome repeats-associated Cas9 nuclease (CRISPR/Cas9) system significantly suppressed the expression of *SMO* and *GLI2*, and reduced Hh signaling in human primary HSCs (pHSCs) and LX-2 HSC lines. *SMO* deletion also blocked *TB4* expression and inactivated HSCs. Furthermore, transgenic mice overexpressing Tb4 (Tb4-Tg) were more susceptible to the development of hepatic fibrosis and showed innate Hh hyperactivity. Taken together, the results suggest that TB4 promoted HSC transdifferentiation by interacting with the Hh signaling pathway.

## Results

### Suppression of TB4 compromised HSC activity

**Figure 1:**
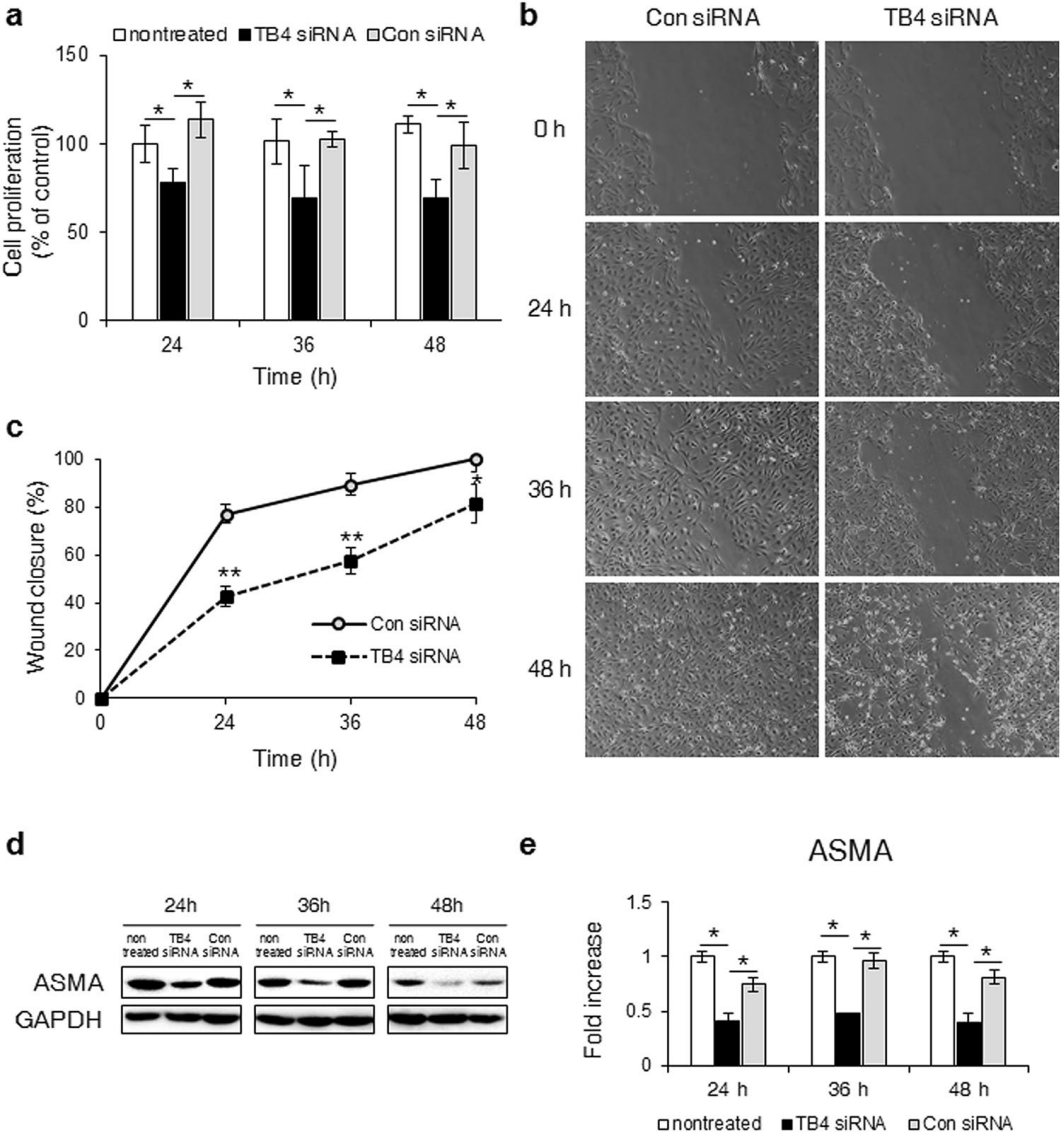
Knockdown of TB4 inhibits the proliferation, migration and activation of HSCs. (**a**) Cell proliferation were measured by MTS assays in LX-2 cells with or without scramble (Con) or TB4 siRNA. The mean ± s.e.m. results obtained from three independent experiments are graphed (**b**,**c**) Cell migration were measured by wound healing assays in Con siRNA- or TB4 siRNA-transfected LX-2 cells. Artificial wounds were created on cells in confluence. Images were taken at 0, 24, 36 and 48 hours after wound. Data shown represent one of three experiments with similar results and the mean ± s.e.m. results obtained from three repetitive experiments are graphed (**b:** Representative images, c: Quantification of wound closure) (**d**,**e**) Protein expression of ASMA in these cells. Data shown represent one of three experiments with similar results and the mean ± s.e.m. results obtained from three repetitive experiments are graphed (GAPDH was used as an internal control) (**d:** Immunoblot, (**e:** Band density) (*p < 0.05; **p < 0.005).

As the activated HSCs expressed higher levels of TB4^25^, we examined whether endogenous TB4 was associated with the functions of HSC. To reduce the level of TB4 in HSCs, we transfected fully activated LX-2, originated from human activated HSC line, with TB4-specific siRNA (TB4 siRNA) for 24, 36, and 48 h. Scrambled siRNA (Con siRNA) was transfected into the LX-2 cells as a negative control. TB4 siRNA efficiently decreased the level of *TB4* expression in the LX-2 cells compared with the Con siRNA- or nontreated cells, as assessed by qRT-PCR and western blot (Supplementary Fig. S1). The number of viable cells in the TB4 siRNA-transfected cells was significantly lower than in the Con siRNA or nontreated cells (Fig. 1a). The reduced expression of *TB4* also inhibited the migration of LX-2 cells compared with the other two groups (Fig. 1b and c). In the TB4 siRNA-treated cells, the expression of alpha smooth muscle actin (ASMA), a marker of HSC activation, gradually decreased during transfection (Fig. 1d and e). These data indicated that the endogenous level of TB4 influenced the viability, migration, and activation of HSCs.

### TB4 interacted with Hh signaling in activated HSCs

**Figure 2:**
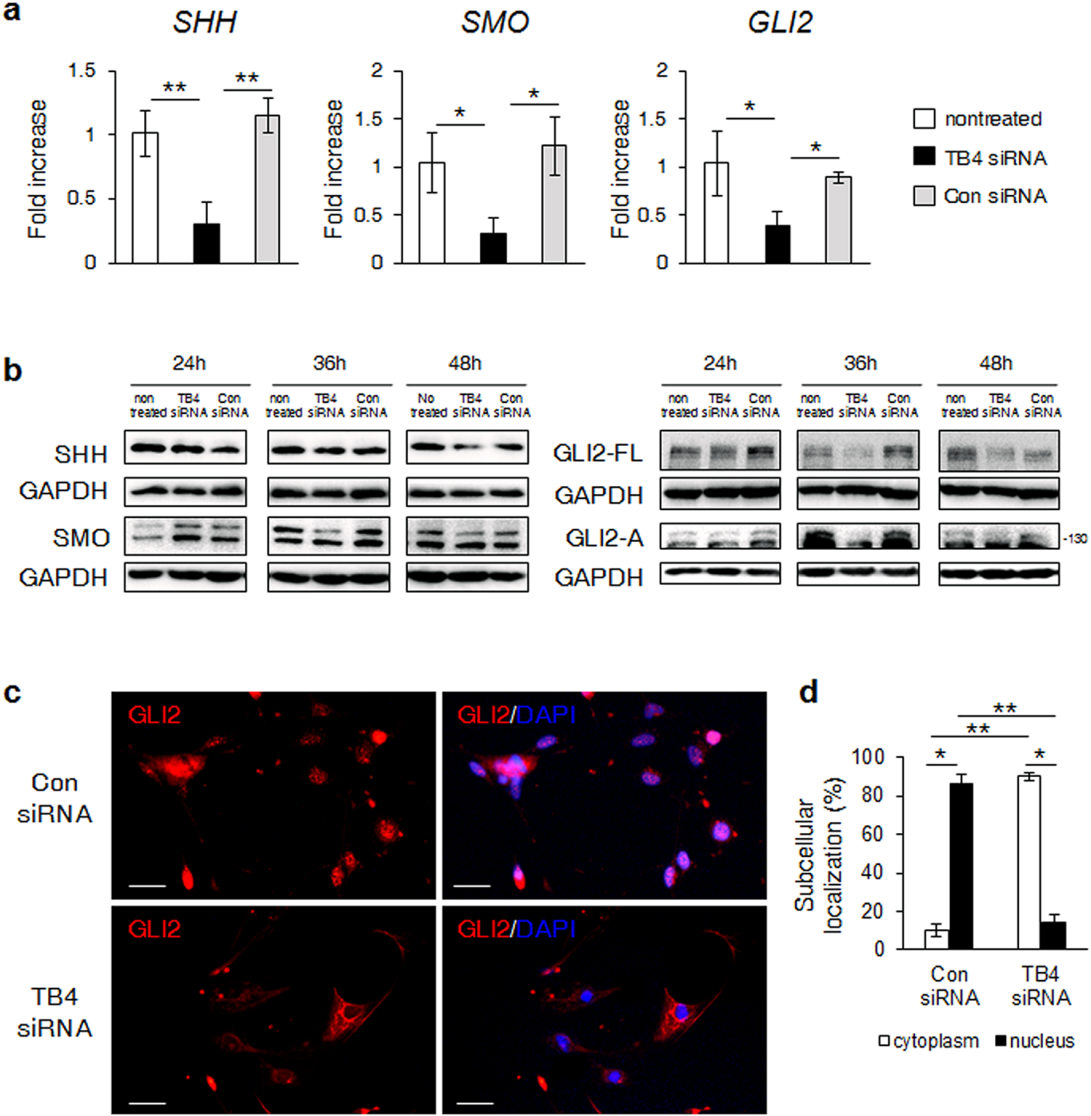
Knockdown of TB4 reduces expression of Hh signaling in HSCs. (**a**) RNA expression of *SHH, SMO* and *GLI2* in nontreated or Con siRNA or TB4 siRNA-transfected LX-2 cells. LX-2 cells were transfected with TB4 or Con siRNA for 24 hours and mRNA levels were analyzed using qRT-PCR. Results of relative expression values are shown as mean ± s.e.m. of triplicate experiments (**b**) Protein expression of SHH, SMO, GLI2-full length (GLI2-FL) and GLI2-activator (GLI2-A) in these cells. Data shown represent one of three experiments with similar results and the mean ± s.e.m. results obtained from three repetitive experiments are graphed (GAPDH was used as an internal control). LX-2 cells were transfected with TB4 or Con siRNA for 24, 36, or 48 hours. Protein levels were analyzed at each time point by immunoblot against the indicated proteins. (**c**) Immunofluorescent staining for GLI2 in the LX-2 cells transfected with Con siRNA or TB4 siRNA. Representative images are shown (scale bar, 100 μm) (×20). (**d**) Quantification of subcellular localization of GLI2 in these cells. The percentage of GLI2-positive cells in nucleus or cytoplasm is shown. (*p < 0.05; **p < 0.005).

As Hh signaling is a well-known pathway regulating HSC activation^12–15^, and our results pointed to an association of TB4 with HSC functions, we investigated whether TB4 was associated with Hh signaling during HSC activation. A qRT-PCR analysis revealed significantly reduced expression of *SHH*, *SMO*, and *GLI2* at the RNA level in the TB4-depleted LX-2 cells 24 h post-transfection compared with the Con siRNA or nontreated cells (Fig. 2a). The protein level of SHH was unchanged at 24 and 36 h but decreased 48 h after transfection of TB4 siRNA compared with the Con siRNA- or nontreated groups. The expression of phosphorylated SMO (active form) was significantly lower in the TB4 siRNA-treated cells than in the other groups at 36 and 48 h. The expression levels of both GLI2-FL and GLI2-A of GLI2 were reduced at 36 and 48 h (Fig. 2b and Supplementary Fig. S2). These results indicated that TB4 depletion induced decreased expression of phosphorylated SMO and GLI2 after 36 h and that the inhibition of these Hh activators subsequently reduced SHH production in HSCs after 48 h, implying that TB4 was closely associated with the SMO-GLI2 axis in Hh signaling.

As GLI2 was down-regulated in the TB4-suppressed LX-2 cells, we assessed the subcellular distribution of GLI2 in these cells. Immunofluorescent staining showed that the GLI2 protein was mostly expressed in the nucleus of the LX-2 cells transfected with Con siRNA (upper lane). In contrast, most of the GLI2 protein was localized in the cytoplasm (lower lane), and its expression was lower in the TB4-transfected cells (Fig. 2c). The quantification data on the subcellular localization of GLI2 revealed significantly higher numbers of cytoplasmic GLI2-expressing cells than nuclear GLI2-expressing cells in the TB4-depleted cells, whereas there were markedly fewer numbers of cytoplasmic GLI2-expressing cells in the control group (Con siRNA-transfected cells; cytoplasmic GLI2-positive cells: 10.05 ± 3.10%, nuclear GLI2-positive cells: 89.95 ± 2.31% per vision field / TB4 siRNA-transfected cells: cytoplasmic GLI2-positive cells 85.99 ± 5.44%, nuclear GLI2-positive cells 14.01 ± 4.59% per vision field) (Fig. 2d). These findings clearly demonstrated that TB4 was involved in the translocation of GLI2 in HSCs.

**Figure 3:**
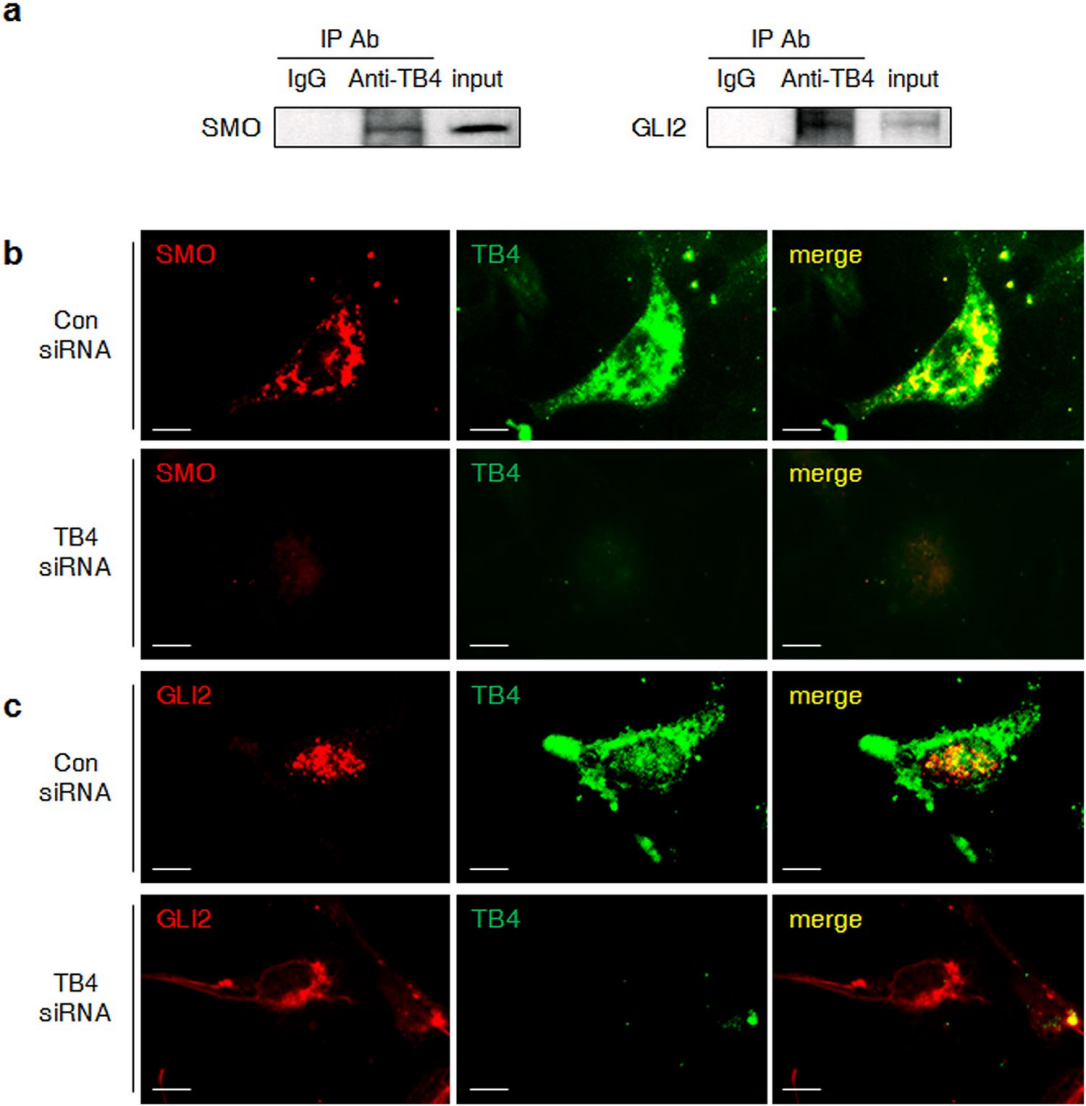
TB4 interacts with either SMO or GLI2 in activated HSCs. (**a**) Immunoblot with SMO or GLI2 in the immunoprecipitated LX-2 lysates with TB4. LX-2 cells were immunoprecipitated with IgG as negative control. 10% of total LX-2 lysates was loaded as input controls. (**b**,**c**) Double immunofluorescent staining for TB4 with SMO (**b**) or GLI2 (**c**) in Con siRNA- or TB4 siRNA-transfected LX-2 cells (×100). Representative images from three experiments with similar results are shown (Scale bar, 20 μm).

Next, the interaction of TB4 with SMO and GLI2 in activated HSCs was examined. As shown by an immunoprecipitation assay, both SMO and the GLI2 protein were detected in anti-TB4 immunoprecipitates from LX-2 cell lysates, suggesting that TB4 interacted with SMO and/or GLI2 in the activated HSCs (Fig. 3a). Double immunofluorescent staining confirmed the interaction between TB4 and SMO and/or GLI2, as observed by a confocal microscope. SMO was colocalized with TB4 in the cytoplasm of the Con siRNA-treated LX-2 cells, whereas neither SMO nor TB4 was detected in the TB4 siRNA-treated cells (Fig. 3b). GLI2 proteins were mostly localized in the nucleus and colocalized with TB4 in the control group. In contrast, they accumulated in the cytoplasm of the TB4-suppressed group, without coexpression of the TB4 protein (Fig. 3c). These results indicated that endogenous TB4 was associated with Hh signaling via an interaction with SMO and GLI2 in HSCs.

### TB4 regulated the SMO-GLI2 axis by activating ILK and phosphorylating GSK3B

**Figure 4:**
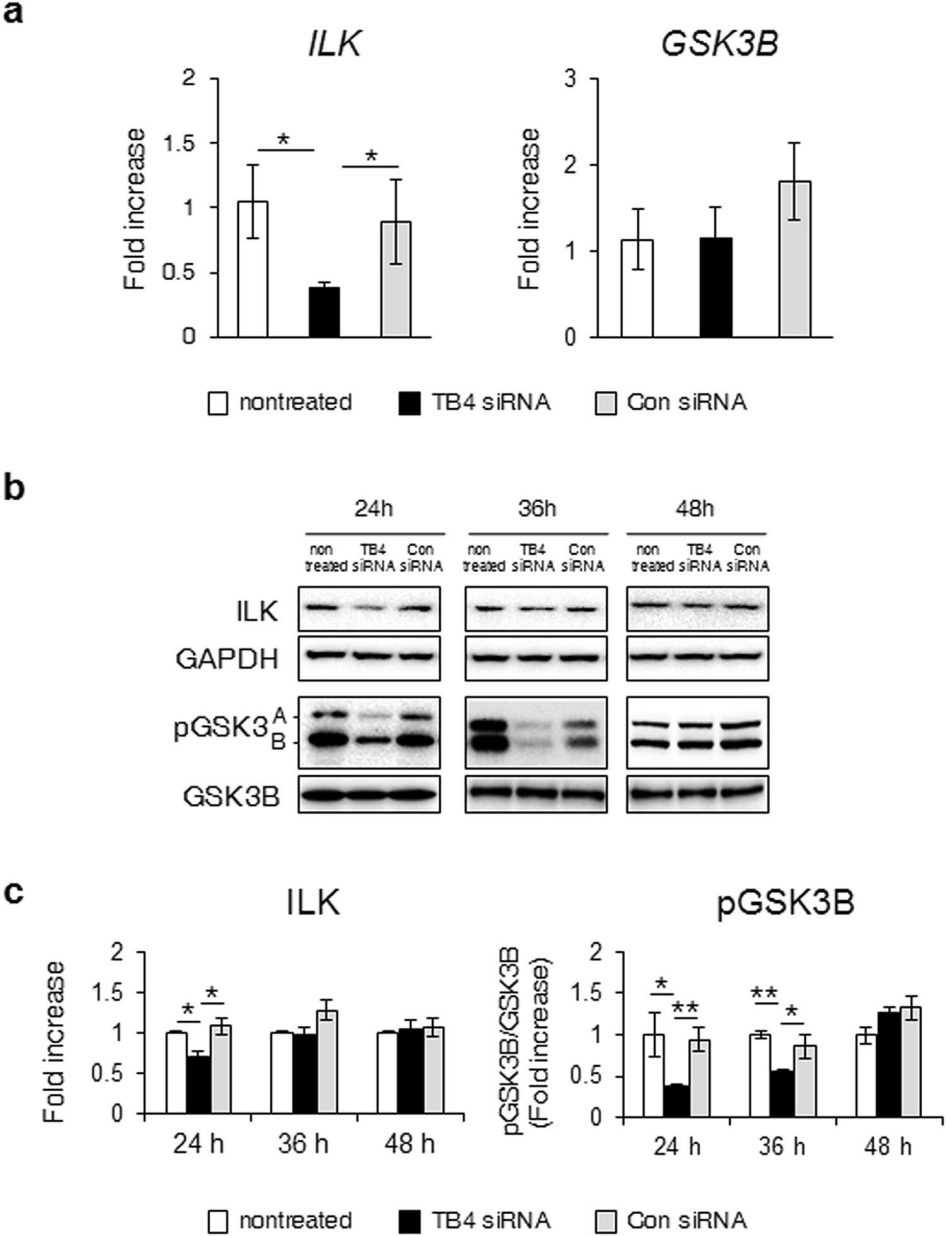
Deficient TB4 blocks expression of ILK and pGSK3B. (**a**) qRT-PCR of *ILK* and *GSK3B* in Con siRNA-, TB4 siRNA- or nontreated LX-2 cells at 24 hours post transfection. Results of relative expression values are shown as mean ± s.e.m. of triplicate experiments (*p < 0.05; **p < 0.005). (**b**,**c**) Protein expression of ILK, pGSK3B and GSK3B in these cells (GAPDH was used as an internal control). Data shown represent one of three experiments with similar results and the mean ± s.e.m. results obtained from three repetitive experiments are graphed (**b:** Immunoblot, (**c:** Band density).

Based on our findings showing the interaction of TB4 with either SMO or GLI2, we further investigated the precise molecular mechanism underlying the involvement of TB4 in regulating the activation of the SMO-GLI2 axis in HSCs. Previous studies reported that ILK-GSK3B regulated GLI2 activation^26–29^ and ILK was shown to be involved in Hh signaling by impacting SMO localization in the cilium^30^. Given the reports that ILK and GSK3B regulated Hh signaling, we examined the association of TB4 with ILK and/or GSK3B in mediating Hh signaling of HSCs. The qRT-PCR analysis showed that the levels of *ILK* decreased in the TB4 siRNA-transfected LX-2 cells after 24 h compared with the Con siRNA- or nontreated LX-2 cells (Fig. 4a). The expression of *GSK3B* at the RNA level did not show the significant changes among these cells. In the TB4-suppressed LX-2 cells, the protein levels of ILK and pGSK3B were down-regulated after 24 h and after 24 and 36 h, respectively (Fig. 4b and c). These results demonstrated that knockdown of TB4 resulted in the reduced expression of ILK and pGSK3B before down-regulating SMO and GLI2 (Fig. 2), suggesting that TB4 was involved in SMO-GLI2 activation by regulating phosphorylation of GSK3B-mediated by ILK during HSC activation.

### CRISPR/Cas9-mediated TB4 knockout inhibited the activation of HSCs by suppressing SMO-GLI2

**Figure 5:**
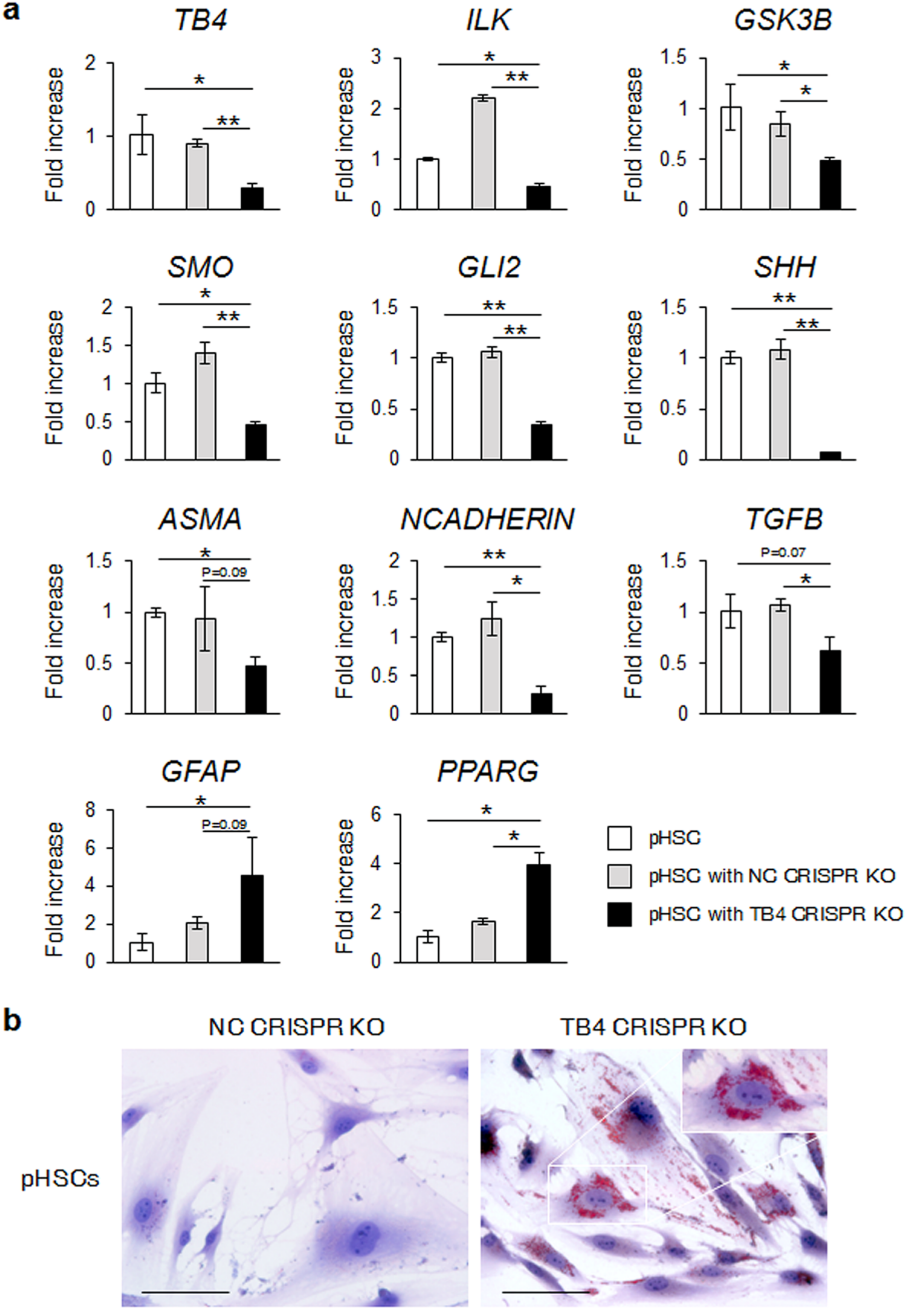
CRISPR/Cas9-mediated TB4 knockout down-regulates Hh expression and promotes inactivation of human primary HSCs. (**a**) qRT-PCR of *TB4, ILK, GSK3B*, Hh signaling (*SMO*, *GLI2*, and *SHH*), markers of HSC activation (A*SMA*, *NCADHERIN*, and *TGFB*) and markers of HSC inactivation (*GFAP* and *PPARG*) in human pHSCs transfected with non-targeting control knockout CRIPSR/Cas9 plasmid (NC CRISPR KO; gray bar) or TB4 knockout CRISPR/Cas9 plasmid (TB4 CRISPR KO; black bar). Expression of these genes expression in nontreated pHSCs was marked as white bar. Results of relative expression values are shown as mean ± s.e.m. of triplicate experiments (*p < 0.05; **p < 0.005.) (**b**) Oil red O staining for lipid droplets in these cells (original magnification ×40, Scale bar, 100 μm). Inserted image presents the magnified image (×63). Representative images from three experiments are shown.

To better understand the Hh-mediated regulatory effects of TB4 on HSC activation in a system in which TB4 was consistently knocked down, we utilized plasmid-based delivery of CRISPR RNA-guided Cas9 nucleases to knock out the *TB4* gene in human pHSCs and LX-2 cells. The TB4 CRISPR/Cas9 knockout plasmid (TB4 CRISPR KO) enabled to specifically cleave TB4. Cells transfected with the nontargeting control CRISPR/Cas9 plasmid (NC CRISPR KO) were established as negative controls. The expression of *TB4* at the RNA level in the TB4 CRISPR KO-treated pHSCs was significantly lower than in the NC CRISPR KO-treated cells. CRISPR/Cas9-mediated depletion of TB4 in the pHSCs decreased the expression of *ILK* and *GSK3B* and Hh signaling molecules, such as *SMO*, *GLI2*, and *SHH*. It also decreased the expression of profibrogenic signaling factors, such as *ASMA*, *NCADHERIN*, and *TGFB*, and increased the level of inactivation markers of HSCs, such as glial fibrillary acidic protein (*GFAP*) and peroxisome proliferator-activated receptor-gamma (*PPARG*), compared with the NC CRISPR KO plasmid-transfected pHSCs (Fig. 5a). Oil Red O staining revealed cytoplasmic lipid droplets, a morphological hallmark of inactivated HSCs, in the human pHSCs transfected with TB4 CRISPR KO, whereas these lipid droplets were rarely detected in the cells transfected with the NC CRISPR KO plasmid (Fig. 5b). These results were also confirmed in the LX-2 cells treated with TB4 and the NC CRISPR KO-transfected LX-2 cells (Supplementary Fig. S3).

### SMO deletion blocked TB4 in HSCs

**Figure 6:**
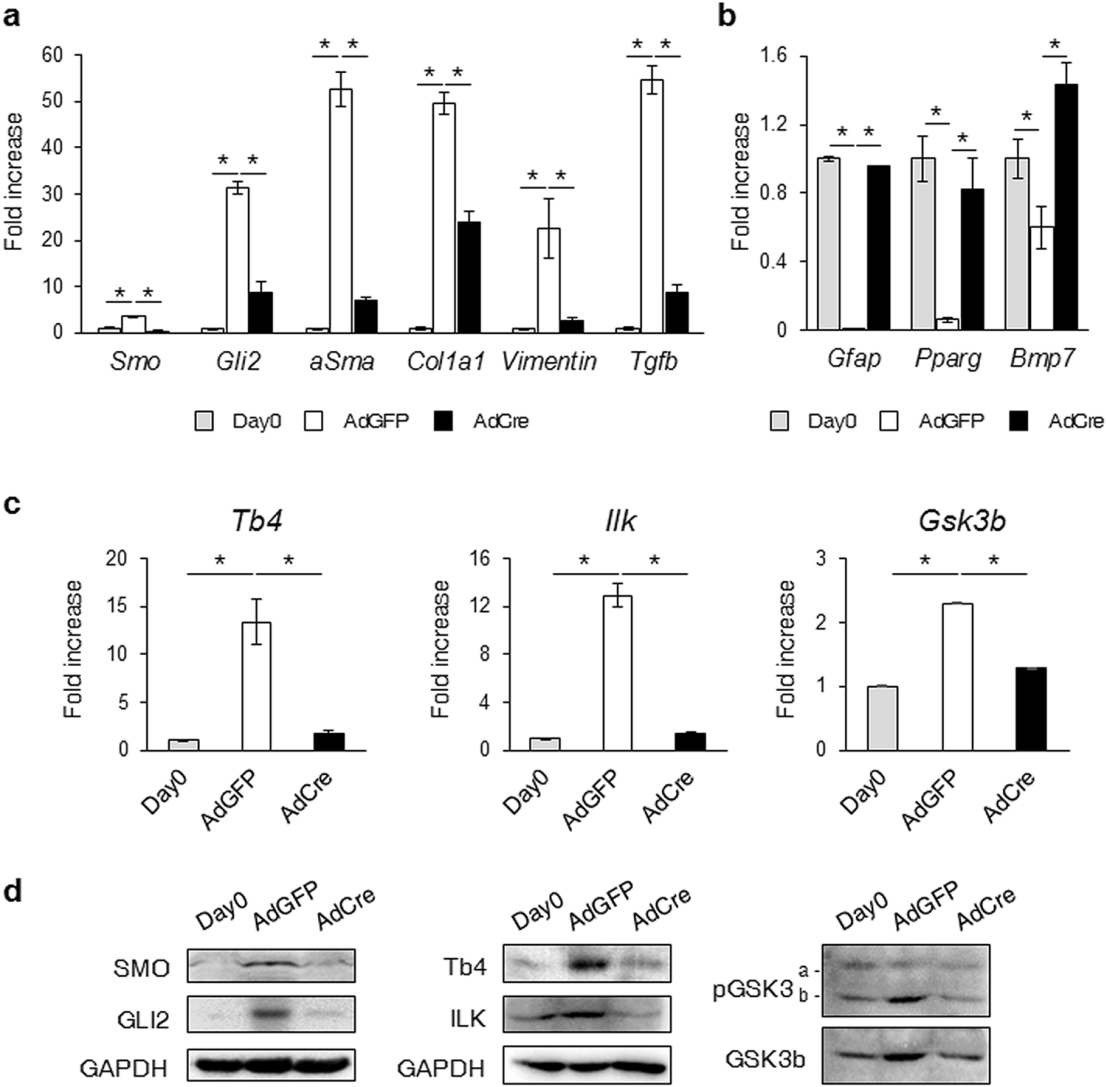
Deleting SMO in HSCs suppresses expression of Tb4, ILK and GSK3b. (**a**) qRT-PCR analysis for Hh signaling (S*mo* and *Gli2*) and markers of activated HSC (*aSma*, *Col1a1*, *Vimentin* and *Tgfb*) in pHSCs at day0 (quiescent stage: white bar) and either AdGFP or AdCre-transfected pHSCs at day 7. These cells are isolated from SMO-flox. (**b**) qRT-PCR analysis for markers of quiescent HSC (*Gfap*, *Pparg* and *Bmp7*) in these cells. (**c**) qRT-PCR analysis for *Tb4*, *Ilk* and *Gsk3b* in these cells. Results are normalized to day 0 HSCs. All results of relative expression values are shown as mean ± s.e.m. of triplicate experiments (*p < 0.05). (**d**) Western blot analysis for SMO, GLI2, Tb4, ILK, pGSK3B and GSK3B in these cells. Data shown represent one of three experiments with similar results (GAPDH was used as an internal control).

As TB4 influenced the activation of SMO and GLI2, pHSCs isolated from SMO-flox mice were treated with adenoviral vectors carrying either Cre recombinase (AdCre) or green fluorescent protein (AdGFP) to examine whether SMO impacted the expression of TB4 in HSCs. Compared with freshly isolated HSCs (day 0), the HSCs treated with AdGFP showed increased expression of *Smo* and *Gli2* and enhanced expression of profibrogenic genes, including *aSma*, collagen1a1 (*Col1a1)*, *Vimentin*, and *Tgfb*, as well as decreased expression of HSC quiescent markers, including *Gfap*, *Pparg*, and bone morphogenetic protein-7 (*Bmp7*). The AdCre-treated HSCs expressed lower levels of *Smo* and profibrogenic genes and higher levels of HSC quiescent markers than the AdGFP-treated HSCs, as assessed by qRT-PCR (Fig. 6a and b). Given that AdGFP did not influence HSC activation and that AdCre efficiently suppressed HSC activation, we next checked the expression of *Tb4*, *Ilk*, and *Gsk3b* in the SMO-disrupted HSCs. The mRNA levels of *Tb4*, *Ilk*, and *Gsk3b* significantly increased in the pHSCs transfected with AdGFP compared with quiescent pHSCs. In contrast, the RNA levels of *Tb4*, *Ilk*, and *Gsk3b* in the pHSCs transfected with AdCre were similar to those of the quiescent pHSCs and greatly decreased compared with those in the AdGFP-treated pHSCs (Fig. 6c). A Western blot analysis also showed reduced expression of SMO, GLI2, TB4, ILK, and pGSK3B in both the quiescent and AdCre-transfected pHSCs compared with the AdGFP-transfected pHSCs (Fig. 6d). In addition, we examined the expression of these genes in LX-2 cells treated with GDC-0449, a SMO antagonist. SMO and its target gene, GLI2, were downregulated 24, 36, and 48 h after the treatment compared with vehicle-treated cells, as assessed by a Western blot analysis. Inactivation of LX-2 cells was confirmed by the reduced expression of ASMA. GDC-0449 also decreased the levels of ILK, pGSK3B, and TB4. Immunofluorescent staining of SMO, GLI2, and TB4 confirmed these results, with remarkably lower detection of these proteins in the LX-2 cells treated with GDC-0449 (Supplementary Fig. S4). These results demonstrated that SMO influenced the expression of TB4, ILK, and GSK3B, implying that TB4 was closely associated with Hh signaling in HSCs.

### Transgenic mice overexpressing Tb4 were more susceptible to the development of hepatic fibrosis

**Figure 7:**
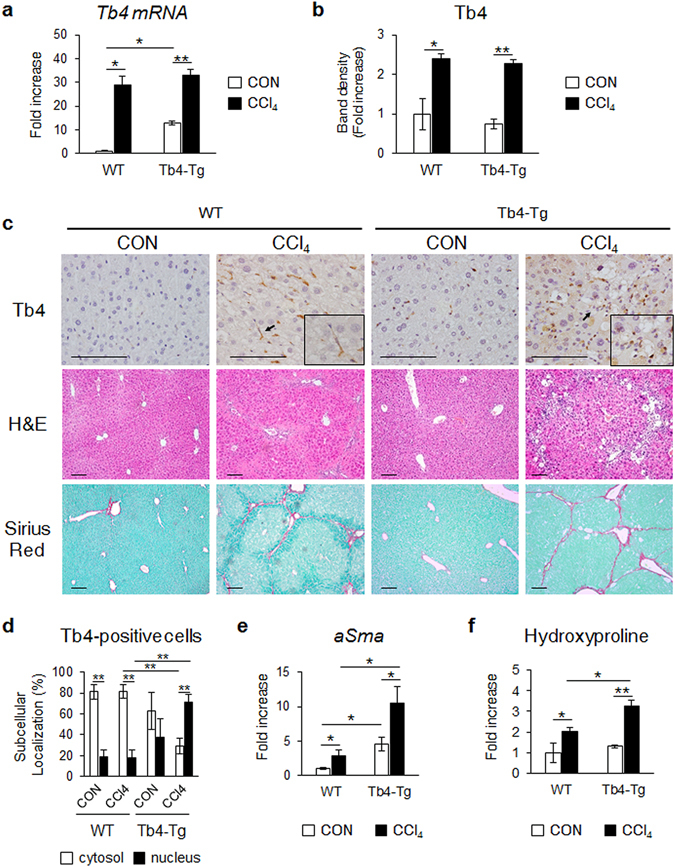
Severe hepatic fibrosis in Tb4 overexpressing transgenic mice. (**a**) qRT-PCR for *Tb4* in livers from wild-type (WT) and Tb4 overexpressing transgenic (Tb4-Tg) mice injected with corn-oil (CON) or carbon tetrachloride (CCl_4_) for 10 weeks (n = 4 mice/group). (**b**) Cumulative densitometric analyses of Tb4 western blot results. (**c**) Representative images of immunostaining for Tb4 (×40), hematoxylin and eosin (H&E) staining (×20) and sirius red staining (×20) in liver sections from representative WT and Tb4-Tg mice with CON or CCl_4._ (scale bar, 100 μm) (**d**) Quantification of nuclear or cytoplasmic Tb4-postive cells in all mice. The number of nuclear or cytosolic Tb4-positive cells were counted in parenchymal area/section (**Nuclear localization p < 0.005). (**e**) RNA expression of *aSma* in livers from all mice. (**f**) Hepatic hydroxyproline contents in liver of all mice. All results are displayed as mean ± s.e.m. (n = 4 mice/group) (*p < 0.05; **p < 0.005).

As the data obtained from the *in vitro* experiments indicated that TB4, acting via SMO and GLI2, was critical to the fate of HSCs, we examined the *in vivo* effect of TB4 on hepatic fibrosis in mice overexpressing the *Tb4* gene (Tb4-Tg). To generate chronic liver fibrosis, wild-type (WT) and Tb4-Tg mice were injected intraperitoneally with CCl_4_ twice a week for 10 weeks. As a control group, equal numbers of mice were treated with corn oil. The level of endogenous *Tb4* was higher in the Tb4-Tg mice than WT mice at both RNA and protein levels. The CCl_4_ injection increased the expression of *Tb4* in both the WT and Tb4-Tg mice, with no difference in *Tb4* expression between the groups, as assessed by qRT-PCR and Western blots (Fig. 7a,b and Supplementary Fig. S5). Immunohistochemical (IHC) staining of Tb4 showed an accumulation of Tb4-positive cells in the fibrotic liver of both groups, as compared with the corn oil-treated own control group. Because the activated HSCs was known to express Tb4^25, 34^, we conducted double immunofluorescent staining for Tb4 and aSMA, in the liver sections of these mice, to examine whether Tb4 was expressed by the activated HSCs in these mice. The double positive cells for aSMA (green color) and Tb4 (red color) were more evident in the CCl_4_-treated mice than the corn oil-treated mice (Supplementary Fig. S6). Interestingly, Tb4 proteins were localized in the cytoplasm or nucleus in the CCl_4_-injected WT mice, whereas they were mostly found in the nucleus in the CCl_4_-injected Tb4-Tg mice (Fig. 7c and d), suggesting that the fibrotic livers of the Tb4-Tg mice contained more active Tb4.

To identity whether an increased expression of TB4 resulted in more severe fibrosis, we examined the liver morphologies and levels of fibrosis in the WT and Tb4-Tg control mice. H&E staining revealed no significant changes between the groups, other than the deposition of a few fatty hepatocytes in the Tb4-Tg control group. The livers of the CCl_4_-injected WT mice showed severe parenchymal damage and centrilobular necrosis compared with the corn oil-injected WT mice. The injured livers of the Tb4-Tg mice also exhibited more severe damage, such as the accumulation of ballooned hepatocytes and necrosis, in addition to more advanced infiltration of immune cells along fibrotic nodules (Fig. 7c H&E and Supplementary Fig. S7). Sirius red staining revealed the accumulation of excessive collagen fibrosis in the CCl_4_-treated livers of the Tb4-Tg mice (Fig. 7c). The RNA level of *aSma* and the hydroxyproline content were higher in the livers of the CCl_4_-treated mice than those of the own control mice, and these fibrotic changes were significantly elevated in the fibrotic livers of the Tb4-Tg mice compared with those of the WT mice treated with CCl_4_ (Fig. 7e and f). These data demonstrated that Tb4-Tg mice were susceptible to the development of fibrosis, indicating that a higher level of *Tb4* contributed to severe hepatic fibrosis.

**Figure 8:**
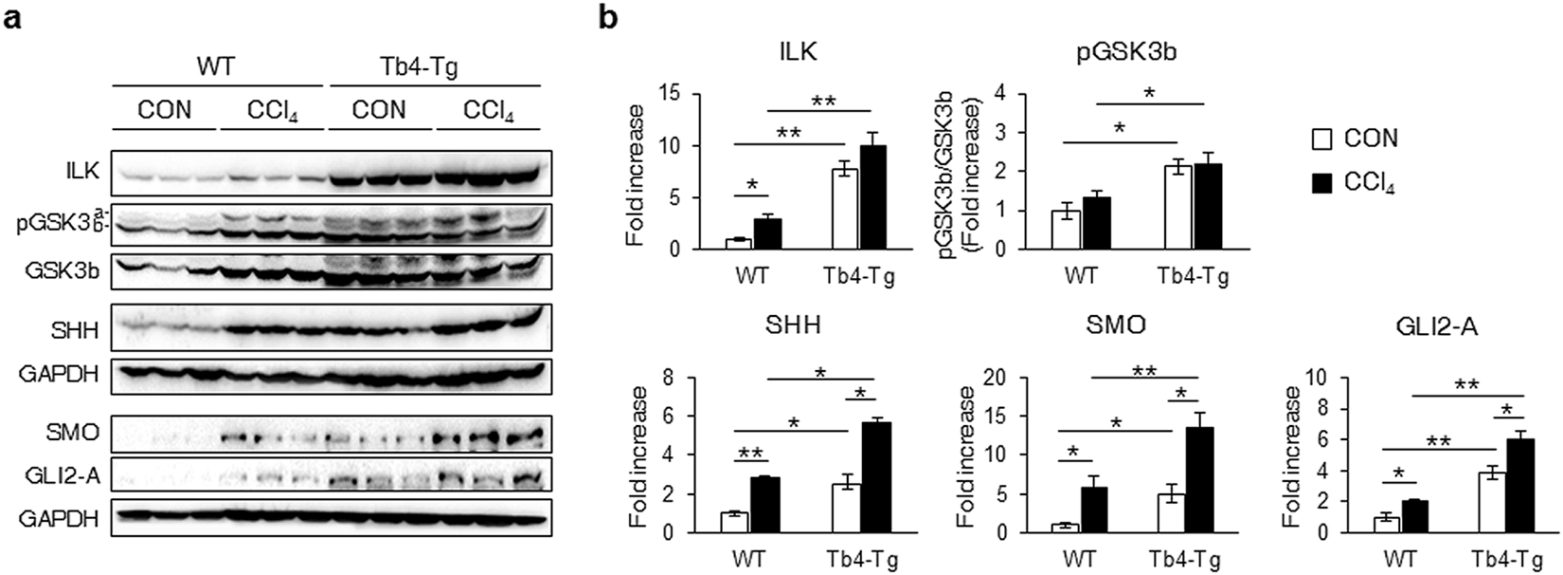
Enhanced expression of Hh, ILK and pGSK3b in livers of Tb4-Tg mice. (**a**) Representative western blot analysis and (**b**) cumulative densitometric analyses of ILK, pGSK3b/GSK3b, SHH, SMO and GLI2-A) in livers from WT and Tb4-Tg mice. (GAPDH was used as an internal control). Immunoblots shown represent one of three experiments with similar results. Results of relative expression are shown as mean ± s.e.m. (*p < 0.05; **p < 0.005).

To investigate whether enhanced expression of *Tb4* increased Hh signaling and contributed to the excessive hepatic fibrosis in the CCl_4_-treated Tb4-Tg mice, we examined the expression levels of ILK, GSK3B and Hh signaling. The baseline expression levels of ILK, GSK3B, SHH, SMO, and GLI2-A were significantly higher in the Tb4-Tg mice than WT mice before injury. After CCl_4_ administration, the expression levels of these genes were elevated compared with those of the livers of the corn oil-treated WT mice. The amount of SHH, SMO and GLI2 increased in the CCl_4_-treated Tb4-Tg mice compared with the levels in the livers of the corn oil-treated Tb4-Tg mice, although the levels of ILK and pGSK3B were not significantly different between both Tb4-Tg groups (Fig. 8a and b). In addition, pHSCs at day 0 after isolation showed the elevated level of Tb4 in the Tb4-Tg mice compared with the WT mice. The pHSCs of Tb4-Tg mice also contained the increased expression of *ILK*, *GSK3B, SMO and GLI2* at the RNA level (Supplementary Fig. S8). These findings indicate that HSCs of the Tb4-Tg mice have the increased activity at baseline. Taken together, these data demonstrated that TB4 overexpression enhanced the activation of SMO-GLI2 and promoted more severe liver fibrosis in mice.

## Discussion

Transdifferentiation of quiescent HSCs to MF-HSCs is pivotal in the pathogenesis of liver fibrosis^4, 5, 13^. Recently, we demonstrated that activated HSCs expressed TB4 in chronically damaged livers and that endogenous expression of TB4 was related with the activation of HSCs^25^. However, it remains unclear how TB4 is involved in regulating HSC transdifferentiation. The present study extends our previous findings by demonstrating that TB4 is closely associated with HSC activation via the Hh signaling pathway, which is one of the key pathways mediating the proliferation and activation of HSCs^12–15^, and that it contributes to hepatic fibrosis.

TB4 is known to promote wound healing, tissue regeneration, and epithelial to mesenchymal transition (EMT) in the cornea, heart, skin, kidney, and nervous system^20, 22, 23, 31, 35–37^. However, the precise molecular mechanism underlying the functions of TB4 remains unknown. Recently, the Cys-His protein (PINCH) and ILK were reported to be intracellular binding partners of TB4^31, 32, 38^. In that report, the researchers demonstrated that TB4 activated ILK by promoting the formation of an ILK and PINCH complex and that this complex mediated the effects of TB4, such as promoting the survival of cardiomyocytes and inhibiting apoptosis of endothelial progenitor cells^31, 38^. In addition, the activation of ILK by TB4 induced EMT in colorectal cancer cells^32^. In damaged liver, ILK was shown to be involved in transmitting fibrogenic signals from the extracellular matrix (ECM) to HSCs and to influence HSC activation and fibrogenesis^39–41^. Shafiei *et al*. reported that ILK expression increased during HSC activation and liver fibrosis^39^. In primary cilia, ILK was reported to be involved in Hh-induced SMO ciliary translocation by interacting with SMO^30^. In the current study, we revealed that either knockdown or knockout of *TB4* in HSCs decreased the expression of *ILK* in HSCs, followed by reduced expression of *SMO*, suggesting that the role of TB4 in SMO activation involved the regulation of ILK expression. However, we did not obtain direct evidence for the formation of an ILK-PINCH complex and subsequent ILK stabilization.

In the present research, knockdown of *TB4* decreased the level of pGSK3B in HSCs, whereas overexpression of *Tb4* increased the level of pGSK3B in the liver of Tb4-Tg mice (Figs 4 and 8). These findings suggested that TB4 contributed to the stabilization of GLI2 via hypo-phosphorylation of GSK3B. TB4 also influenced the level of phosphorylated GSK3 alpha (GSK3A), an isoform of GSK3, in HSCs (Fig. 4). GSK3A was hyper-phosphorylated in the livers of the Tb4-Tg mice compared to those of the WT mice before and after CCl_4_ treatment (Fig. 8). GSK3A and GSK3B have been reported to exert different functions, despite the very high sequence similarity (98%) of their catalytic domains^42, 43^. For example, GSK3B decreased both hypertrophy and heart failure, whereas GSK3A increased these symptoms in heart pressure overload^42^. In pancreatic cancer, GSK3A but not GSK3B induced constitutive noncanonical NF-kappa-B signaling by stabilizing nuclear p52, although upregulation of GSK3A and GSK3B caused by mutant K-ras resulted in activation of constitutive canonical NF-kappa-B^43^. Recent findings also demonstrated that GSK3A was involved in hepatic glucose metabolism^44^, indicating that GSK3A and GSK3B exhibited divergent physiological roles in the liver. In support of these findings, in the present study, the phosphorylation level of GSK3A was significantly higher in the Tb4-Tg mice than WT mice after the CCl_4_ treatment, although the expression level of pGSK3B was unchanged in both groups, pointing to the possibility that GSK3A is involved in regulating liver fibrosis, with or without the cooperation of GSK3B. Further studies are needed to identify the potential role of GSK3A in the liver.

In the present study, in an experimental animal model of chronic liver disease, we showed that Tg mice overexpressing *Tb4* were vulnerable to extensive hepatic fibrosis in response to chronic CCl_4_ exposure. Other research showed that genetically altered mice with an overly active Hh pathway accumulated more MF-HSCs and developed worse liver fibrosis after bile duct ligation than littermate controls^45^. Thus, increased Hh activity due to the upregulation of *Tb4* is a reasonable explanation for the marked fibrosis in the CCl_4_-treated Tb4-Tg mice. After the transfection of TB4 siRNA in HSCs, the ILK protein was down-regulated after 24 h, and the level of pGSK3B was decreased after both 24 and 36 h. All protein level of SMO, GLI2-FL and GLI2-A declined in the HSCs after 36 and 48 h. Given that GSK3B is one of the targets of ILK^29, 46^, it is possible that TB4-dependent activation of ILK and concomitant repression of pGSK3B increases Hh signaling by promoting the activation of SMO-GLI2, resulting in the activation of quiescent HSCs during liver fibrosis. It is well established that activated HSCs play an important role in the development of liver fibrosis/cirrhosis^4–6^. Excessive HSC activation may lead to abnormal ECM deposition, resulting in progressive fibrosis. In the current study, removal of TB4 by RNA interference-mediated gene silencing or CRISPR/Cas9-mediated gene knockout was sufficient to inactivate HSCs through inhibition of Hh signaling, specifically SMO-GLI2. Furthermore, either genetic or pharmacological inhibition of *SMO* in HSCs resulted in a dramatic reduction of *TB4*. In addition, upregulation of *Tb4* in the Tb4-Tg mice enhanced SMO activation and GLI2 stabilization and activation. Although the protein level of TB4 in the Tb4-Tg mice was similar before and after injury, the increased number of nuclear TB4 protein in the Tb4-Tg mice indicates increased activity of *Tb4*. Since SMO-GLI2 axis plays an essential role in regulating HSC activation^14, 16, 19^, the inactivated Hh signaling pathway caused by SMO suppression leads HSC inactivation^12, 16–18^. Tb4 reduction seems to be accompanied during HSC inactivation, because Tb4 is also required to activate HSCs^25^. In line with this possibility, Tb4 deletion suppresses both ILK-mediated SMO activation and SMO-regulated GLI2 activation, thereby contributing to down-regulation of SMO and GLI2 during HSC inactivation. Therefore, these findings strongly support our hypothesis that TB4 is involved in regulating the fate of HSCs through Hh signaling pathway and suggest that TB4 may be a potential target in regulating Hh signaling.

Either the canonical or noncanonical Hh signaling pathway ultimately exerts its effects by modulating the activation of Glis^47–49^. Given that GLI2 has a dominant role in pathological processes of liver fibrosis^9, 19^, understanding of the stabilization and activation of GLI2 is important. Herein, we demonstrated, for the first time, that TB4 was a key regulator of GLI2 activation. TB4 interacted with GLI2 in the activated HSCs (Fig. 3a). Deletion of *TB4* using either siRNA or CRISPR/Cas9 suppressed the expression of GLI2 in HSCs (Figs 2a and 5a). HSCs treated with TB4 siRNA contained fewer nuclear GLI2-postive cells than the cells treated with Con siRNA, suggesting the possibility that TB4 might be involved in the nuclear translocation of GLI2 in HSCs. The involvement of TB4 in the activation of GLI2 and its association with SMO in HSCs were also demonstrated by the interaction of TB4 with SMO, in addition to GLI2, and by the fact that its expression influenced SMO expression and vice versa. In addition, the *in vivo* effects of *Tb4* on *Gli2* were confirmed by the upregulation of SMO and GLI2-A in cases of advanced fibrosis in the Tb4-Tg mice. Therefore, TB4 seems to be involved in canonical Hh signaling. However, further studies are required to investigate the roles of TB4 in canonical and noncanonical Hh signaling.

Growing evidence suggest that Tb4 is translocated into the nucleus, although it has been known as the major G-actin-sequestering protein in the cytoplasm^50–54^. In addition, Tb4 was reported to influence the expression of several biological effectors, such as vascular endothelial growth factor, TGF-b, laminin-5 and runt-related transcription factor 2^55–57^. Tb4 has been suggested to modulate the functions of transcription factors rather than acting as transcription factor itself^58, 59^, because it does not have the DNA-binding or transactivation domain which is found in the typical transcriptional factors^60^. In human corneal epithelial cell line HCET, Tb4 was shown to be colocalized with transcription factor RelA/p65, a subunit of NF-kappa-B transcription complex, in the cytoplasm. In the response to TNF-a stimulation, Tb4 was translocated into the nucleus with p65, and inhibited p65 from binding to the promoter of interleukin-8^58^. In line with these findings, we showed the colocalization of Tb4 with Gli2 in the activated HSC nuclei. Our i*n vivo* studies also revealed that the CCl_4_-injected Tb4-Tg mice having more Tb4 in the nucleus and less Tb4 in the cytoplasm showed the significant increase of Hh signaling compared with the CCl_4_-injected WT mice. Therefore, these results suggest the potential of Tb4 as a novel regulator modulating the action of transcriptional factor, Gli2, in HSCs.

In conclusion, the present study demonstrated that TB4 enhanced the activation of SMO-GLI2 by regulating the expression of ILK and pGSK3b, as well as interacting with SMO and GLI2, thereby promoting HSC activation and liver fibrosis. These findings shed light on the mechanism underlying the effects of TB4 in liver fibrosis and point to the potential role of TB4 as a novel therapeutic target in the development of treatments for chronic liver disease.

## Methods

### Experimental animals

Smo^tm2Amc^/J (SMO-flox) mice were purchased from The Jackson Laboratory and male WT C57BL/6 mice were purchased from Hyochang (Dae-gu, Korea). Male Tb4-Transgenic (Tb4-Tg) mice generated on C57BL/6 background as described previously^61^ were gift from Dr. Moon (Sejong University, Seoul, Korea). These mice were housed with 12-h light/dark cycle and allowed free access to normal food and water. Mice at 7 weeks of age were used for all the experiments. To induce liver fibrosis, 7-week-old WT mice (n = 5) and Tb4-Tg mice (n = 4) received 0.6 ml kg^−1^ body weight of CCl_4_ (Sigma-Aldrich, St Louis, MO, USA) dissolved in corn oil by intraperitoneal injection, twice a week for 10 weeks^62^. As a control, same number of mice was injected with equal volume of corn oil. All mice were sacrificed, to obtain serum and liver sample, at 48 h post the last injection of CCl_4_ or corn oil. All animal care and surgical procedures were carried out according to the provisions of the National Institutes of Health (NIH) guidelines for the care and Use of Laboratory. The animal protocol used in this study has been approved by the Pusan National University–Institutional Animal Care and Use Committee (PNU-IACUC) on their ethical procedures and scientific care, and it has been approved (Approval Number PNU-2016-1286).

### TB4 siRNA transfection

LX-2, human HSC line (provided by Dr. Jeong, KAIST, Korea), was cultured in DMEM (Gibco, Life Technologies, Carlsbad, CA, USA) supplemented with 10% fetal bovine serum (FBS, Gibco, Life Technologies) and 1% penicillin/streptomycin (P/S, Gibco, Life Technologies) at 37 °C in a humidified atmosphere containing 5% CO_2_. As determined by trypan blue exclusion, cell viability was >92% in all experiments. To knockdown of TB4 in LX-2 cells, LX-2 cells (1 × 10^5^ per well) were transfected with 25 nM of TB4 siRNA (ON-TARGETplus SMARTpool, Dharmacon Inc., Chicago, IL, USA) or 25 nM of Con siRNA (ON-TARGETplus Non-targeting Pool, Dharmacon Inc.) as a negative control using Lipofectamine RNAiMAX transfection reagent (Invitrogen, Life Technologies, Carlsbad, CA, USA) according to the manufacturer’s instruction. These cells were harvested at 24, 36 or 48 hours post transfection of siRNA. To inhibit SMO, fully activated LX-2 cells were treated with SMO antagonist Vismodegib (1 μM of GDC-0449; Selleck Chemicals, Houston, TX, USA) or vehicle (DMSO, Sigma-Aldrich) for 24, 36 and 48 hours.

### CRISPR/Cas9-mediated TB4 knockout in human HSCs

To generate TB4 knockout LX-2 cells or human pHSCs (purchased from Zen-Bio Inc., NC, USA), CRISPR/Cas9 mediated TB4 knockout was done according to the manufacturer’s instructions (Santa Cruz Biotechnology, Inc., CA, USA). TB4 CRISPR/Cas9 KO plasmid (Santa Cruz Biotechnology, Inc.) consisting of a pool of 3 plasmids is designed to disrupt gene expression by causing a double-strand break in a 5′ constitutive exon within the TMSB4X gene. The control CRISPR/Cas9 Plasmid (Santa Cruz Biotechnology, Inc.) containing a non-targeting 20 nt scramble guide RNA (gRNA) was used as a negative control. Briefly, cells (1 × 10^5^ cells per well) were seeded onto 6-well culture plates in 3 ml of antibiotics-free DMEM per well, 24 hours prior to transfection and grown to 70% confluency. Cells were transfected with 1 μg of TB4 CRISPR/Cas9 KO Plasmid and TB4 HDR Plasmid (Santa Cruz Biotechnology, Inc.) using UltraCruz® Transfection Reagent (Santa Cruz Biotechnology, Inc.) and incubated at 37 °C, 5% CO_2_. Two days after transfection, successful co-transfection of the CRISPR/Cas9 KO Plasmid and HDR Plasmid was visually confirmed by detection of the red fluorescent protein via an Olympus IX71 fluorescence microscope (Olympus Optical Co., Ltd. Tokyo, Japan). The medium was changed with fresh medium containing puromycin (0.5 μg/ml; Santa Cruz Biotechnology, Inc.) to allow the selection of stably transfected cells with successful integration. Cells were incubated with puromycin for at least 72 hours and then cultured in regular medium for downstream experiments.

### Isolation of primary HSCs from mice

Primary HSCs were isolated as described previously^16^. Briefly, WT C57BL/6, Tb4-Tg and SMO-flox male mice were anaesthetized with zoletil 50 (5 mg kg^−1^ body weight, Virbac SA, France), to immobilize in the recumbent position on a treatment table, and the inferior vena cava was cannulated under aseptic conditions. Livers were perfused *in situ* with EGTA and collagenase (Roche, Indianapolis, IN, USA), to disperse the cells. Primary HSCs were isolated by differential centrifugation on OptiPrep (Sigma-Aldrich) density gradient and located on the upper layer of 11.5% OptiPrep. The purity of HSCs was >98%, as established by microscopy examination for lipid droplets and vitamin A auto-fluorescence. Isolated HSCs were seeded at a density of 3 × 10^2^ cells/mm^2^ and cultured in RPMI 1640 medium (Gibco, Life Technologies) containing 10% FBS and 1% P/S at 37 °C in a humidified atmosphere containing 5% CO_2_.

### Adenoviral transfection in primary HSCs isolated from mice

HSCs isolated from SMO-flox male mice were cultured for 3 days, and then starved in serum-depleted medium for overnight. Adenoviruses harboring either the GFP gene (AdGFP, Vector Biolabs, Malvern, PA, USA) or Cre recombinase gene (AdCre, Vector Biolabs) were added to these pHSCs at a multiplicity of infection (MOI) of 80, as described previously^16^. After 24 hours, virus-containing medium was aspirated and replaced with fresh medium. Viral efficiency of AdGFP infection was assessed by fluorescent microscope, with >95% of infected cells found to be GFP positive.

### Cell Proliferation (MTS) assay and Scratch assay

Cell proliferation was measured with a CellTiter Proliferation Assay (Promega, Madison, WI, USA) as described in previous report^63^. Briefly, cells were plated at a density of 1 × 10^4^ cells per well in 96-well plates with the indicated treatment and time. After adding the MTS reagent, the plates were incubated in a CO_2_ incubator at 37 °C until the color developed. Absorbance was then measured at a wavelength of 490 nm using a Glomax multi-detection system (Promega). Standard wound healing assays were performed by growing cells to a confluent monolayer, and making a manual scratch using a 200 μL pipette tip. Then, floating cells were washed out and fresh medium was added. As the incubation time passed, the width of scratch was narrow and it was recorded by taking photographs (×20) using an Olympus inverted microscope (Olympus Optical Co., Ltd.) from 24, 36 and 48 hours after the scratch. Empty area in each time point was quantified with NIH image J version 1.49 analysis software (Rasband, W.S., ImageJ, U.S. National Institutes of Health, Bethesda, Maryland, USA, http://imagej.nih.gov/ij/, 1997–2012) and compared with that in the initiation of cell migration.

### Hydroxyproline assay

Hydroxyproline content of the livers was calculated by the method previously described^62^. Briefly, 50 mg of freeze-dried liver tissue was hydrolysed in 6 N HCL at 110 °C for 16 h. The hydrolysate was evaporated under vacuum and the sediment was re-dissolved in 1 ml distilled water. Samples were filtered using 0.22 μm filter centrifuge tube at 14,000 r.p.m. for 5 minutes. Lysates were then incubated with 0.5 ml of chloramines-T solution containing 1.41 g of chloramine-T dissolved in 80 ml of acetate–citrate buffer and 20 ml of 50% isopropanol, at room temperature. After 20 minutes, 0.5 ml of Ehrlich’s solution, containing 7.5 g of dimethylaminobenzaldehyde dissolved in 13 ml of 60% perchloric acid and 30 ml of isopropanol, was added to the mixture, which was incubated at 65 °C for 15 minutes. After cooling to the RT, the absorbance was read at 561 nm. Amount of hydroxyproline in each sample was determined using the regression curve from the hydroxyproline prepared with high purity hydroxyproline (Sigma-Aldrich) and divided by the amount of liver weight contained in the initial sample (50 mg) to get the hydroxyproline contents (μg of hydroxyproline per mg of liver tissue). Data were expressed as fold changes by comparing with hydroxyproline content of the control group.

### RNA analysis

Total RNA was extracted from liver tissues or cells by using TRIzol reagent (Ambion, Life Technologies). The concentration and purity of RNA were determined using a nanodrop. Template complementary DNA was synthesized from total RNA using the SuperScript First-strand Synthesis System (Invitrogen, Life Technologies) according to the manufacturer’s protocols. We performed the real-time qRT–PCR analysis by using Power SYBR Green Master Mix (Applied Biosystem, Life technologies) on the manufacturer’s specifications (Eppendorf, Mastercycler Real-Time PCR). All reactions were triplicated and data were analysed according to the ΔΔCt method. 40S ribosomal protein S9 mRNA and 18S ribosomal RNA were used for normalization of the expression level. The sequences of all primers used in this study are summarized in Supplementary Table S1. All PCR products were directly sequenced for genetic confirmation (Macrogen, Inc., Seoul, Korea).

### Western Blotting

Total protein was extracted from frozen liver tissues stored at −80 °C and cultured cells. Samples were homogenized in triton lysis buffer supplemented with protease inhibitors (Roche) and centrifuged at 13,000 r.c.f. for 15 minutes. The supernatants containing protein extracts were used in subsequent biochemical analysis. Protein concentration was measured by Pierce BCA Protein Assay Kit (Thermo Scientific, Waltham, MA, USA). Equal amount of total protein lysates was separated by SDS–polyacrylamide gel electrophoresis (PAGE) on 8 or 10% tris-glycine gel according to the method of Laemmli, and then transferred onto a 0.45-μm pore size polyvinylidene difluoride (PVDF) membrane (Millipore, Darmstadt, Germany). For TB4 detection, tris-tricine SDS-PAGE was performed according to Schägger and Von Jagow^64^ using 15% tris-tricine gel and other detailed procedures were conducted as previously described^21^. The anode buffer consisted of 0.2 M Tris-Hcl (pH 8.9) and the cathode buffer consisted of 0.1 M Tris-Hcl, 0.1 M Tricine and 0.1% SDS (pH 8.25). After SDS-PAGE, the gels were incubated for 1 h in PBS containing 10% glutaraldehyde (Sigma-Aldrich), washed three times for 20 min in PBS, incubated in a transfer buffer containing 20% methanol for 30 min at room temperature and then transferred onto a 0.2-μm pore size PVDF membrane (Invitrogen). Primary antibodies used in this study were as follows: rabbit anti-TB4 antibody (diluted 1:5000; Immundiagnostik AG, Bensheim, Germany), rabbit anti-SHH antibody (diluted 1:100; Santa Cruz Biotechnology, Inc.), rabbit anti-SMO antibody (diluted 1:1,000; Abcam, Cambridge, MA, USA), rabbit anti-GLI2 antibody (diluted 1:1,000; GenWay Biotech, Inc., San Diego, CA, USA), rabbit anti-pGSK3A/B antibody (diluted 1:1,000; Cell Signaling Technology, Inc., Danvers, MA, USA), rabbit anti-GSK3B antibody (diluted 1:1,000; Cell Signaling Technology, Inc.), mouse anti-ILK antibody (diluted 1:1,000; BD Biosciences, San Diego, CA, USA), mouse anti-ASMA antibody (diluted 1:1,000; Sigma-Aldrich), and mouse anti-glyceraldehydes 3-phosphate dehydrogenase antibody (GAPDH) (diluted 1:1,000; AbD Serotec, Oxford, UK) as an internal control. Horseradish peroxidase-conjugated anti-rabbit or anti-mouse IgG (Enzo Life Sciences, Inc., Farmingdale, NY, USA) was used as secondary antibody. Protein bands were detected using an EzWestLumi ECL solution (ATTO Corporation, Tokyo, Japan) as per the manufacturer’s specifications (ATTO Corporation, Ez-Capture II). Densities of protein bands were measured using CS Analyzer software (Version 3.00.1011, ATTO & Rise Corporation).

### Immunoprecipitation assay

For immunoprecipitation assay, protein (400 μg) was immunoprecipitated overnight with mouse anti-TB4 primary antibody (Novus Biologicals, Littleton, CO, USA) and protein G Plus/Protein A agarose beads (Calbiochem, Darmstadt, Germany) as described in previous report^65^. They were washed three times with lysis buffer and boiled in 5× sample buffer for 10 minutes followed by centrifugation. Resulting clear supernatants were subjected to SDS-PAGE. The following steps were taken using the same method applied for Western blotting.

### Immunofluorescence

Immunofluorescence was carried out as previously described^25^. Briefly, cultured HSCs on coverslips were transfected with siRNA for 36 h, fixed in and permeabilized with cold acetone and methanol, respectively. Cells were washed with PBS and incubated with blocking solution (Dako, Carpinteria, CA, USA) for 30 minutes. Cells were incubated with primary antibody for 4 °C overnight. Cells were washed in PBS and incubated with fluorescein labelled secondary antibodies for 1 hour at room temperature. For double staining, cells were washed in PBS, incubated with blocking solution for 10 minutes and then incubated with second primary antibody for 3 hours at room temperature. Cells were washed in PBS and incubated fluorescein labelled secondary antibodies for 1 hour at room temperature. The following primary antibodies were used: Rabbit anti-SMO antibody (diluted 1:100; Abcam), rabbit anti-GLI2 antibody (diluted 1:100; GenWay Biotech, Inc.), mouse anti-ASMA antibody (diluted 1:200; Sigma-Aldrich), mouse anti-TB4 antibody (diluted 1:100; Novus Biologicals), and rabbit anti-TB4 antibody (diluted 1:100; Immundiagnostik AG). The following secondary antibodies were used: Alexa Fluor 568 goat anti-rabbit IgG (diluted 1:100; Invitrogen) or Alexa Fluor 488 chicken anti-mouse IgG (H + L) (diluted 1:100; Invitrogen). Slides were mounted on slides antifade mounting medium with 4′,6-diamidno-2-phenylinole (DAPI, VectaShield, Burlingame, CA, USA). Slides were viewed with a Zeiss LSM 510 confocal microscope (Carl Zeiss Inc., Thornwood, NY, USA) or an Olympus IX71 fluorescence microscope (Olympus Optical Co. Ltd). For quantitative analysis for localization of Gli2, Gli2-positive cells in nuclear or cytoplasmic localization were counted.

### Liver histology and immunohistochemical staining

To examine hepatic morphology and assess liver fibrosis, H&E staining and Sirius red staining were performed, respectively. Liver specimens were fixed in 10% neutral buffered formalin (Sigma-Aldrich), embedded in paraffin, cut into 4 μm sections and placed on glass slide. Next, sections were deparaffinized with xylene, hydrated with ethanol, and stained by standard methods performed as previously described. For immunohistochemistry, liver sections were deparaffinized, hydrated and incubated in 3% hydrogen peroxide to block endogenous peroxidase. Antigen retrieval was performed by heating in 10 mM sodium citrate buffer (pH 6.0) for 10 min using microwave. Sections were blocked in Protein Block solution (Dako) for 30 min at room temperature followed by incubation with rabbit anti-TB4 antibody (diluted 1:8000; Immundiagnostik AG) at 4 °C overnight. Other sections were also incubated at 4 °C overnight in non-immune sera. Polymer-horseradish peroxidase anti-rabbit (Dako) was used as secondary antibody and 3,30- diaminobenzidine as brown color was used to visualize the protein. Tissue sections were counter-stained with Hematoxylin (Sigma-Aldrich). To quantify the number of TB4-positive cells, 10 areas (zone 2 area) of the histological slides were randomly selected per section at ×40 magnification. The number of nuclear or cytoplasmic TB4-positve cells were counted as cells positive for TB4 possessing a clear nucleus (visualized by hematoxylin counterstaining).

### Oil Red O staining

Staining of LX-2 cells and primary HSCs was performed as previously described^25, 66^. Cultured cells on coverslips were fixed with 4% paraformaldehyde in PBS for 15 min. Cells were stained with Oil Red O (0.5% in propylene glycol, Sigma-Aldrich) revealing lipid droplets. Nuclei was counterstained with hematoxylin for light microscopic examination.

### Statistics

Results are expressed as the mean ± s.e.m. Statistical significances were determined by the unpaired two-sample Student’s t-test. Differences were considered as significant when P-values are <0.05. Statistical analyses were performed using IBM SPSS Statistics 21 software (Release version 21.0.0.0, IBM Corp., Armonk, NY, USA).

### Data Availability

All data generated or analysed during this study are included in this published article and its Supplementary Information files.

## Electronic supplementary material

Supplementary information.
