# Supplementary material for: Thymosin beta-4 regulates activation of hepatic stellate cells via hedgehog signaling
Source: Sci Rep. 2017 Jun 19;7:3815. doi: 10.1038/s41598-017-03782-x (PMC5476572; doi:10.1038/s41598-017-03782-x)
Supplement: Supplementary file 1 — Supplementary information. [file 41598_2017_3782_MOESM1_ESM.pdf]

Supplementary Information to

Thymosin beta-4 regulates activation of hepatic stellate cells via Hedgehog signaling

Jieun Kim, Jeongeun Hyun, Sihyung Wang, Chanbin Lee, Jae-Wook Lee, Eun-Yi Moon,

Heejae Cha, Anna Mae Diehl and Youngmi Jung*

Table of Contents

Supplementary Table ………………………..………………………..……………….…… 2

Supplementary Figure ………..…………..…………………………..…………………..…. 4

Supplementary Table S1. Primer sequences used for real-time qRT-PCR. Primer sequences shown in this table were used for real-time qRT-PCR. All values were normalized to the level of 9S/18S rRNA for total mRNA.

Gene	Forward sequence	Reverse Sequence
hsa ***SHH***	AGT GGC CAG GAG TGA AAC TG	GCC CTC GTA GTG CAG AGA CT
hsa ***GFAP***	CTG GAG GTT GAG AGG GAC AA	CAG CCT CAG GTT GGT TTC AT
hsa ***VIMENTIN***	CGA AAA CAC CCT GCA ATC TT	GTG AGG TCA GGC TTG GAA AC
hsa ***TGFB***	GGG AAA TTG CTC GAC GAT	TTG ACT GAG TTG CGA TAA TGT T
hsa ***SMO***	CTG GTG TGG TTT GGT TTG TG	AGA GAG GCT GGT AGG TGG TG
hsa ***NCADHERIN***	CCA TCA CTC GGC TTA ATG GT	ACC CAC AAT CCT GTC CAC AT
hsa ***GLI2***	CGT GGT GCA GTA CAT CAA GG	CAG AGA AGC CAG TGC TT CC
hsa ***COL1A1***	TGT GAG GCC ACG CAT GAG	CAG ATC ACG TCA TCG CAC AA
hsa ***ASMA***	CTT TTC CAT GTC GTC CCA GT	GTG ACG AAG CAC AGA GCA AA
hsa ***S9***	CTT CAT CTT GCC CTC GTC CA	GAC TCC GGA ACA AAC GTG AGG T
hsa ***TB4***	CGC AGA CCA GAC TTC GCT CGT AC	TCC TTC CCT GCC AGC CAG ATA GAT
hsa ***ILK***	AAG GTG CTG AAG GTT CGA GA	ATA CGG CAT CCA GTG TGT GA
hsa ***GSK3B***	GGA ACT CCA ACA AGG GAG CA	TTC GGG GTC GGA AGA CCT TA
hsa ***PPARG***	CGT GGC CGC AGA TTT GAA	CTT CCA TTA CGG AGA GAT CCA C
mmu ***Shh***	GGA ACT CAC CCC CAA TTA CA	TGC ACC TCT GAG TCA TCA GC
mmu ***Smo***	CAG CAA GAT CAA CGA GAC CA	AAG TGG CAG CTG AAG GTG AT
mmu ***Bmp7***	GTG GTC AAC CCT CGG CAC A	GGC GTC TTG GAG CGA TTC TG
mmu ***aSma***	AAA CAG GAA TAC GAC GAA G	CAG GAA TGA TTT GGA AAG GA
mmu ***S9***	GAC TCC GGA ACA AAC GTG AGG T	CTT CAT CTT GCC CTC GTC CA
mmu ***Vimentin***	GCT TCT CTG GCA CGT CTT GA	CGC AGG GCA TCG TTG TTC

**mmu *Gli2***	CAA GCA GAA CAG CGA GTC AG	CCT CAG CCT CAG TCT TGA CC
**mmu *Ncadherin***	CAG TGG ACA TCA ATG GCA ATC A	CAT TTG GAT CAT CCG CAT CA
**mmu *Col1a1***	GAG CGG AGA GTA CTG GAT CG	GCT TCT TTT CCT TGG GGT TC
**mmu *Gfap***	GCT TCC TGG AAC AGC AAA AC	ATC TTG GAG CTT CTG CCT CA
**mmu *18S***	CTG GAT ACC GCA GCT AGG AA	CCC TCT TAA TCA TGG CCT CA
**mmu *Tgfb***	TTG CCC TCT ACA ACC AAC ACA A	GGC TTG CGA CCC ACG TAG TA
**mmu *Tb4***	ATG TCT GAC AAA CCC GAT ATG GC	CCA GCT TGC TTC TCT TGT TCA/
**mmu *Ilk***	GTGAATGAGCACGGCAATGTG	CCCATTTTCTCTGCCCGTTCT
**mmu *Pparg***	CAG GAG AGC AGG GAT TTG CA	CCT ACG CTC AGC CCT CTT CAT
**mmu *Gsk3b***	CAG GGC ACC AGA GTT GAT CTT	GCT CCC TTG TTG GTG TTC CTA

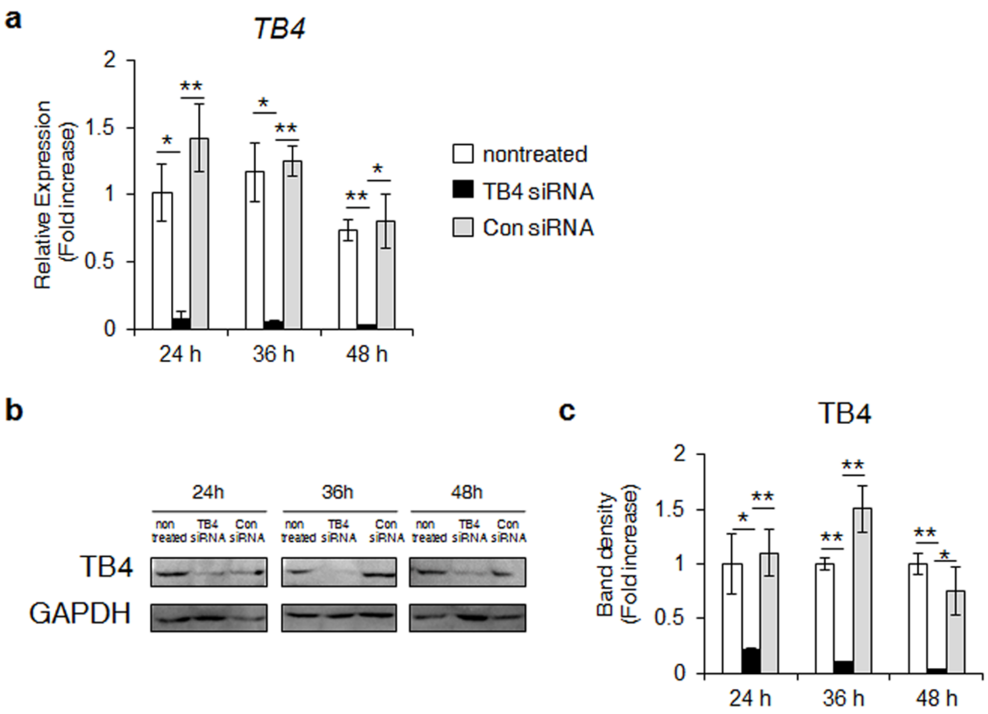

Supplementary Fig. S1. TB4 expression in TB4 siRNA-treated LX-2 cells

(a) qRT-PCR for *TB4* in LX-2 cells with or without Con siRNA or TB4 siRNA. Results of relative expression values are shown as mean±s.e.m. of triplicated experiments (*p<0.05; **p<0.005). (b and c) Western blot analysis of TB4 in these cells (GAPDH was used as an internal control). Data shown represent one of three experiments with similar results (b: Immunoblot, c: Band density). Results of relative expression values are shown as mean±s.e.m. of triplicate experiments (*p<0.05; **p<0.005).

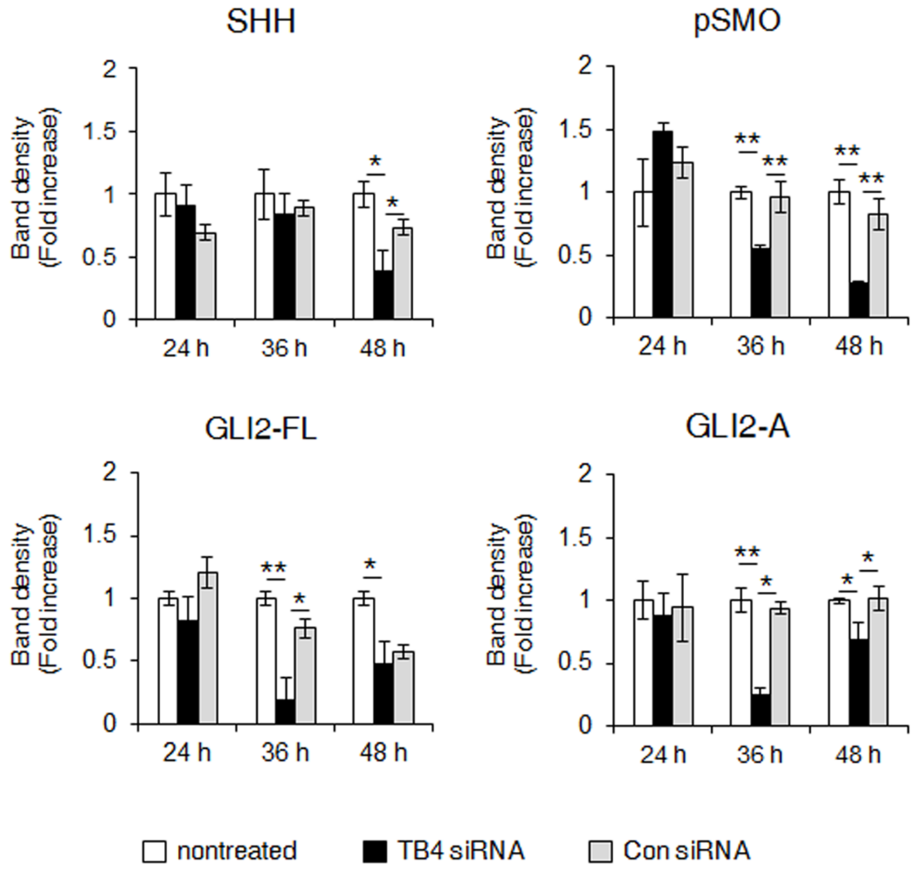

Supplementary Fig. S2. Protein expression of SHH, SMO and GLI2 in LX-2 cells transfected with scramble or TB4 siRNA.

Cumulative densitometric analyses of SHH, SMO, GLI2-FL and GLI2-A western blotting results are displayed as the mean±s.e.m. of triplicate experiments (*$p<0.05$; **$p<0.005$).

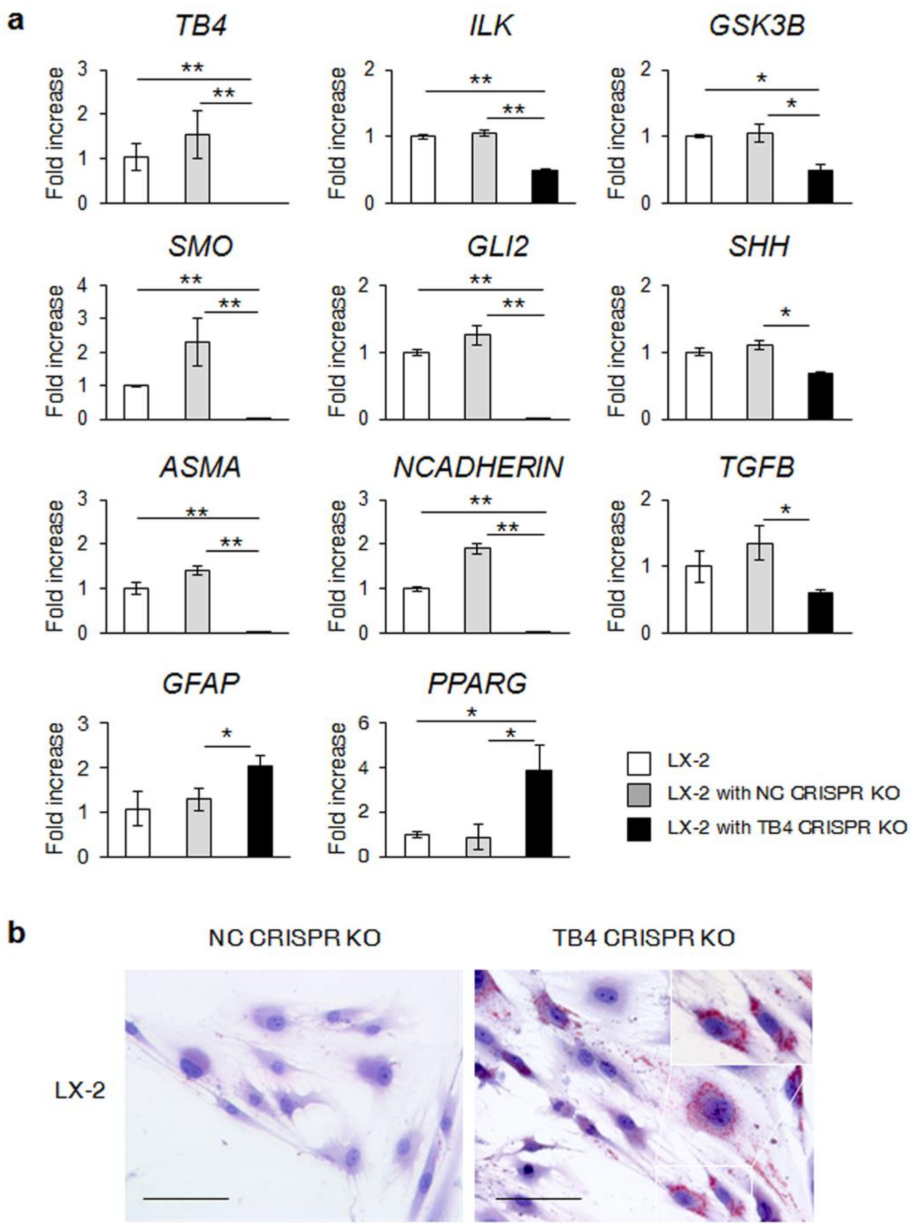

Supplementary Fig. S3. Deleting TB4 by CRISPR/Cas9 suppresses Hh signaling and inactivates LX-2 cells.

(a) qRT-PCR of *TB4*, *ILK*, *GSK3B*, Hh signaling (*SMO*, *GLI2*, and *SHH*), markers of HSC activation (*ASMA*, *NCADHERIN*, and *TGFB*) and markers of HSC inactivation (*GFAP* and *PPARG*) in LX-2 cells transfected with non-targeting control knockout CRIPSR/Cas9 plasmid

(NC CRISPR KO; gray bar) or TB4 knockout CRISPR/Cas9 plasmid (TB4 CRISPR KO; black bar). Expression of these genes expression in nontreated LX-2 cells was marked as white bar. Results of relative expression values are shown as mean±s.e.m. of triplicate experiments (*p<0.05; **p<0.005.) (b) Oil red O staining for lipid droplets in these cells (original magnification x40, Scale bar, 100μm). Inserted image shows the magnified images (x63). Representative images from three experiments are shown.

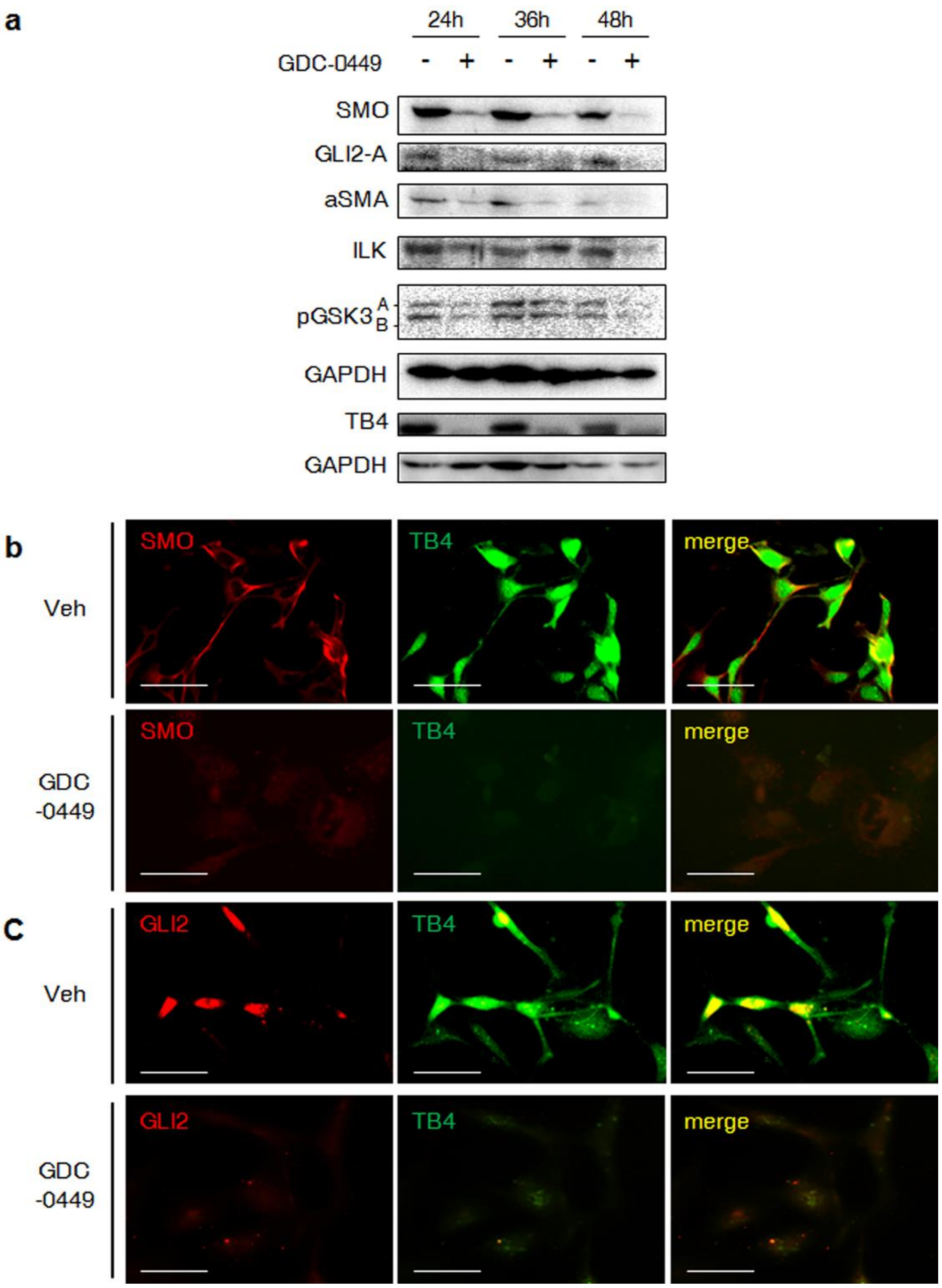

Supplementary Fig. S4. SMO antagonist decreases the expression of TB4, ILK and pGSK3B in LX-2 cells.

(a) Western blot analysis for SMO, GLI2-A, ASMA, ILK, pGSK3B and TB4 in LX-2 cells treated

with 1μM of GDC-0449, a smoothened (SMO) antagonist, for 24, 36 and 48 hours. Data shown represent one of three experiments with similar results. (GAPDH was used as an internal control.) (b and c) Double immunofluorescent staining for TB4 with SMO (b) or GLI2 (c) in these cells (x40, scale bar, 100μm). Representative images from three experiments with similar results are shown.

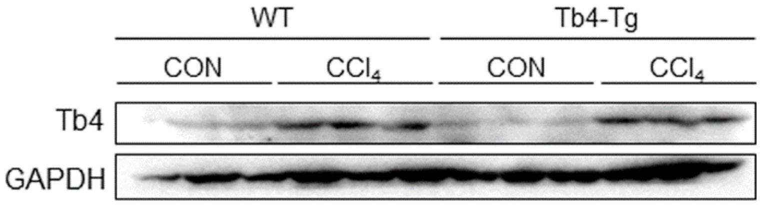

Supplementary Fig. S5. Protein expression of Tb4 in livers of WT and Tb4-Tg mice injected with corn-oil or CCl_4.

Western blot analysis for Tb4 in livers of the representative mice from WT and Tb4-Tg mice injected with corn-oil or CCl_4 for 10 weeks (n=3 / group). Data shown represent one of three experiments with similar results (GAPDH was used as an internal control).

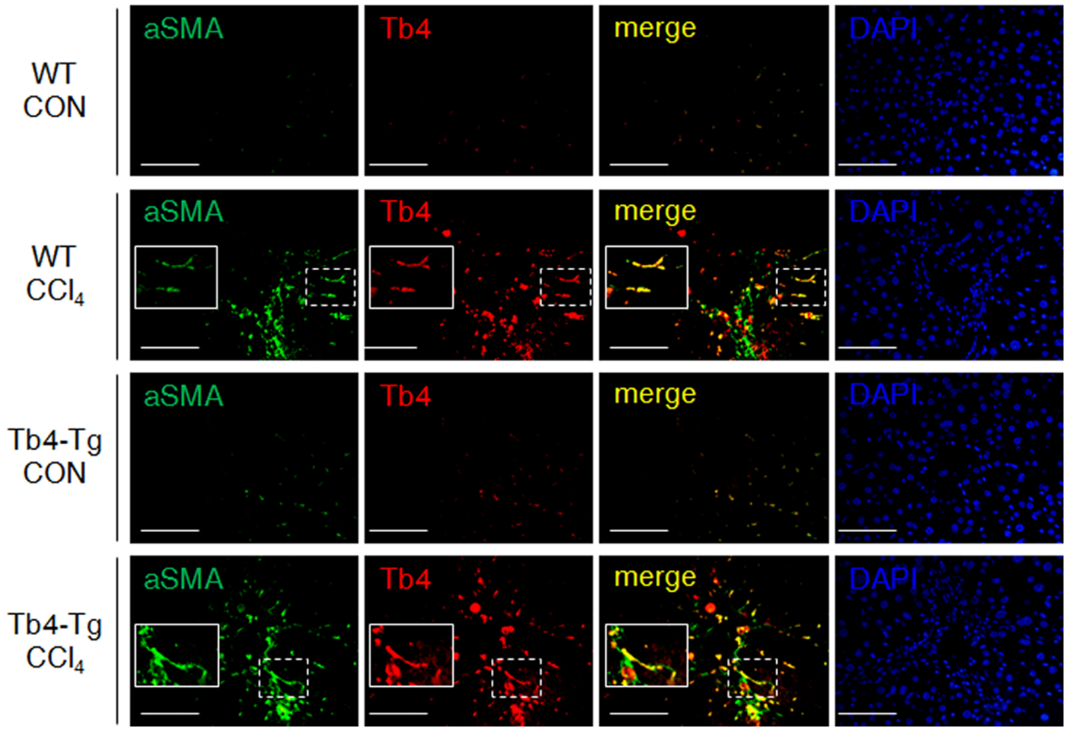

Supplementary Fig. S6. Co-localization of Tb4 and aSMA in the damaged livers.

Double immunofluorescent staining for Tb4 and aSMA in liver section from representative wild-type (WT) and Tb4-overexpressing transgenic (Tb4-Tg) mice injected with either corn-oil (CON) or carbon tetrachloride (CCl_4)-treated mice (x 40). Red and green colors indicate Tb4 and aSMA, respectively. Co-expressing cells are shown as yellow to orange. DAPI nuclear staining is shown as blue. Inserted images presents the magnified images (x 63). (scale bar, 100μm)

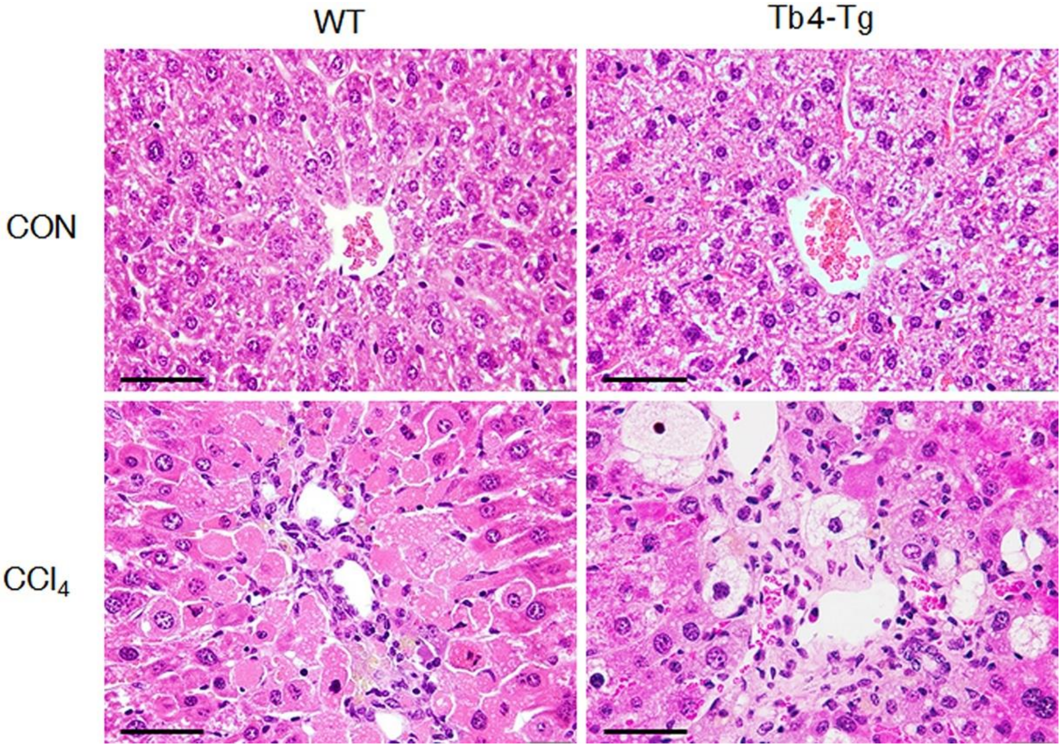

Supplementary Fig. S7. Histomorphology in livers of WT and Tb4-Tg mice injected with corn-oil or CCl_4.

H&E stained liver sections from representative wild-type (WT) and Tb4-overexpressing transgenic (Tb4-Tg) mice injected with either corn-oil (CON) or carbon tetrachloride (CCl_4). (scale bar, 100μm)

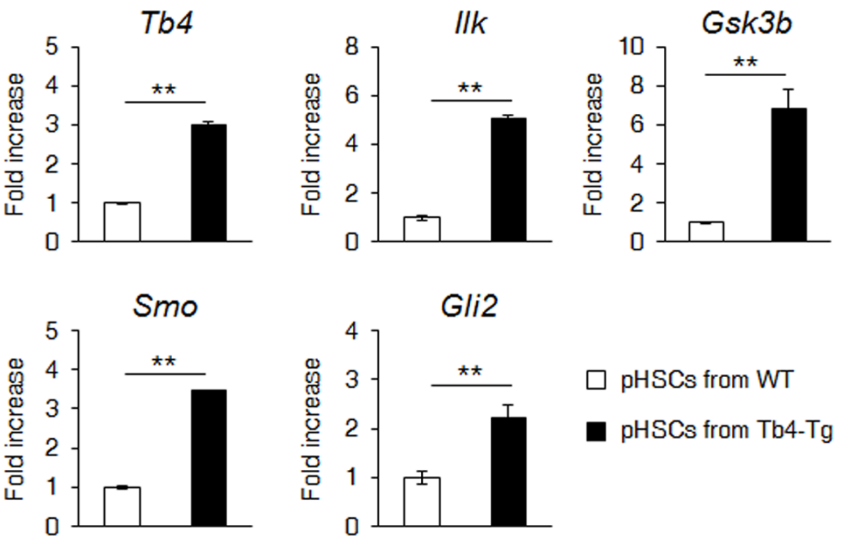

Supplementary Fig. S8. Increased expression of Tb4, Ilk, Gsk3b, Smo and Gli2 in primary HSCs of Tb4-Tg mice

QRT-PCR analysis of *Tb4*, *Ilk*, *Gsk3b*, *Smo* and *Gli2* in primary HSC (pHSCs) isolated from the wild-type (WT; white bar) and Tb4-overexpressing transgenic (Tb4-Tg; black bar) mice. Mean±s.e.m. results are graphed (*p<0.05; **p<0.005).
